# Supplementary material for: Proximity-labeling proteomics reveals remodeled interactomes and altered localization of pathogenic SHP2 variants
Source: EMBO Rep. 2025 Dec 22;27(3):793–826. doi: 10.1038/s44319-025-00674-4 (PMC12894930; doi:10.1038/s44319-025-00674-4)
Supplement: Supplementary file 1 — Appendix [file 44319_2025_674_MOESM1_ESM.pdf]

Appendix Figures for:

Proximity-labeling proteomics reveals remodeled interactomes and altered localization of pathogenic SHP2 variants

Anne E. van Vlimmeren[1,2], Lauren C. Tang[2], Ziyuan Jiang[1], Abhishek Iyer[2], Rashmi Voleti[1], Konstantin Krismer[3], Jellert T. Gaublomme[2], Marko Jovanovic[2], Neel H. Shah[1,4,*]

[1] Department of Chemistry, Columbia University, New York, NY 10027

[2] Department of Biological Sciences, Columbia University, New York, NY 10027

[3] Koch Institute for Integrative Cancer Research, Massachusetts Institute of Technology, Cambridge, MA

[4] Herbert Irving Comprehensive Cancer Center, Columbia University, New York, NY 10032

* corresponding author: neel.shah@columbia.edu

Table of contents:

Appendix Figure S1. Affinity-purification mass spectrometry with SHP2^{WT} and SHP2^{T42A}. pg 2
Appendix Figure S2. STRING interaction networks for core SHP2 interactomes. pg 3
Appendix Figure S3. Effects of biotin on mitochondrial TurboID signal. pg 4
Appendix Figure S4. SHP2 proximity-labeling distributions in subcellular compartments. pg 5
Appendix Figure S5. AlphaFold 3 models of the SHP2-PPIF interaction. pg 6

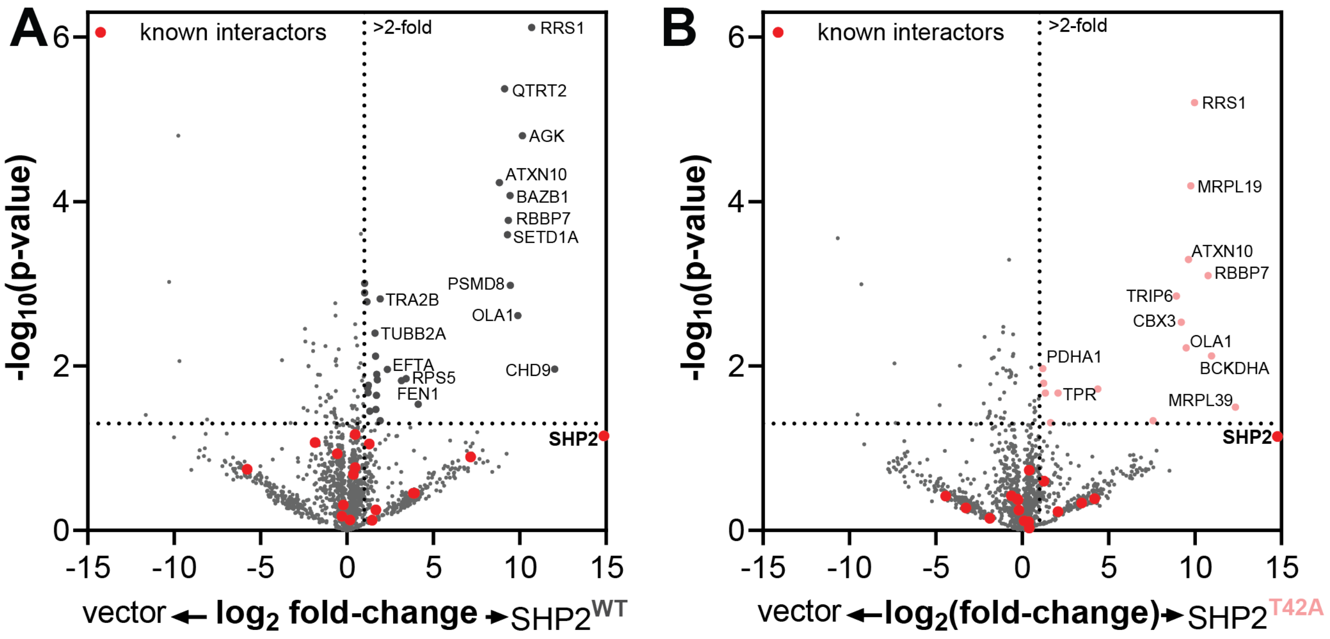

Appendix Figure S1. Affinity-purification mass spectrometry with SHP2^{WT} and SHP2^{T42A}. (**A**) Volcano plot showing proteins enriched in SHP2^{WT} over a vector control using affinity purification mass spectrometry. No known positives were identified (n = 3 biological replicates). P-values were determined using a heteroscedastic, two-tailed t-test. (**B**) Same as (**A**), but for SHP2^{T42A} (n = 3 biological replicates).

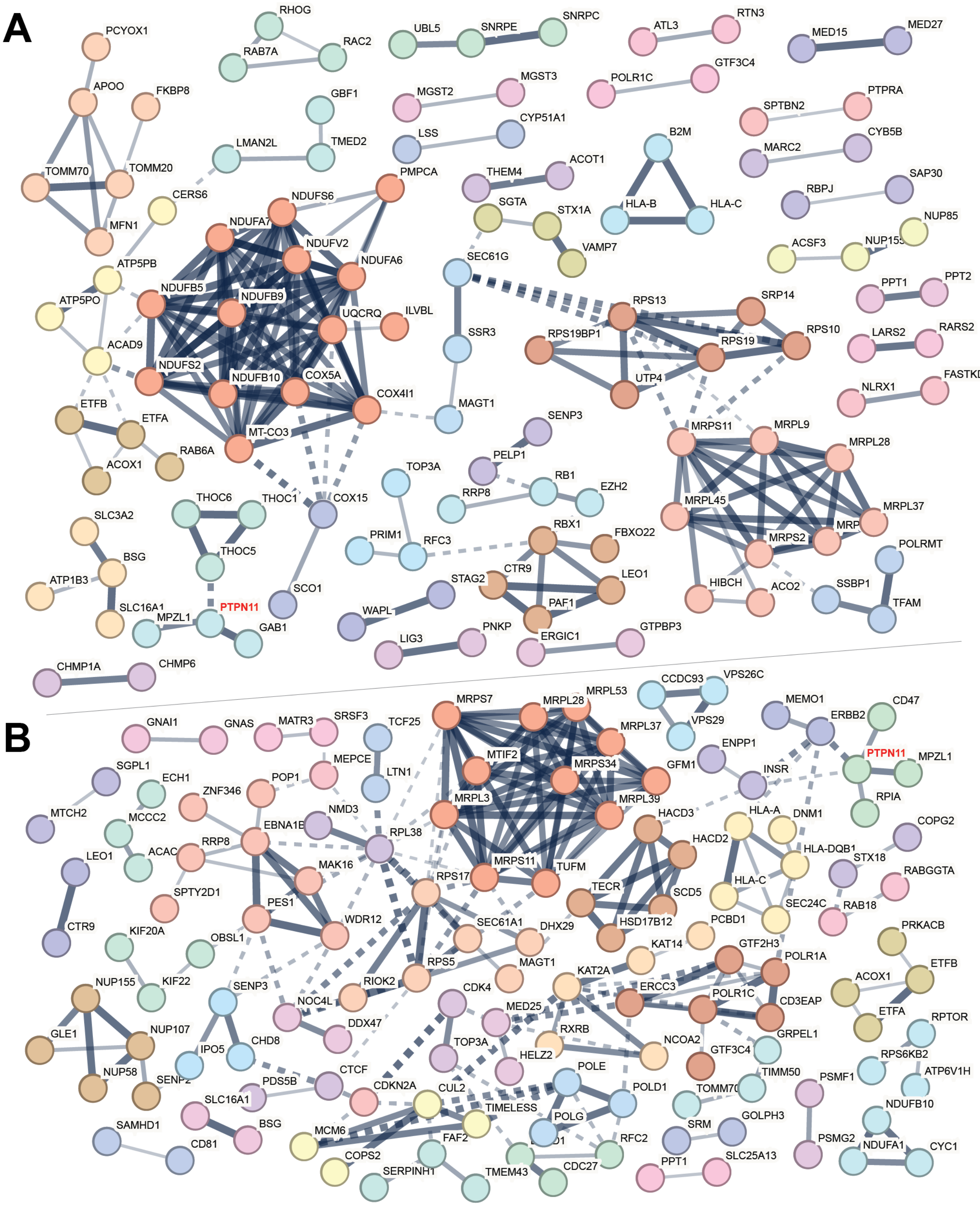

Appendix Figure S2. STRING interaction networks for core SHP2 interactomes. (**A**) STRING interaction network for proteins significantly enriched over the TurboID-only control by 4 or more SHP2 variants in the absence of EGF stimulation. (**B**) STRING interaction network for proteins significantly enriched by 3 or more SHP2 variants in the absence of EGF stimulation. For both panels, significant enrichment is defined as fold-change >2, p-value <0.05 (heteroscedastic, unpaired t-test). Solid lines between proteins indicate a known physical interaction between proteins within a cluster, as documented in the STRING database. Dashed lines also indicate a known physical interaction, but for proteins between two clusters. Only proteins that have a physical interaction with at least one other protein in our interactomes and have an edge confidence score of at least 0.4 are shown. Edge thickness represents edge confidence: thin = 0.4, medium = 0.7, thick = 0.9. Clusters were identified by Markov Clustering.

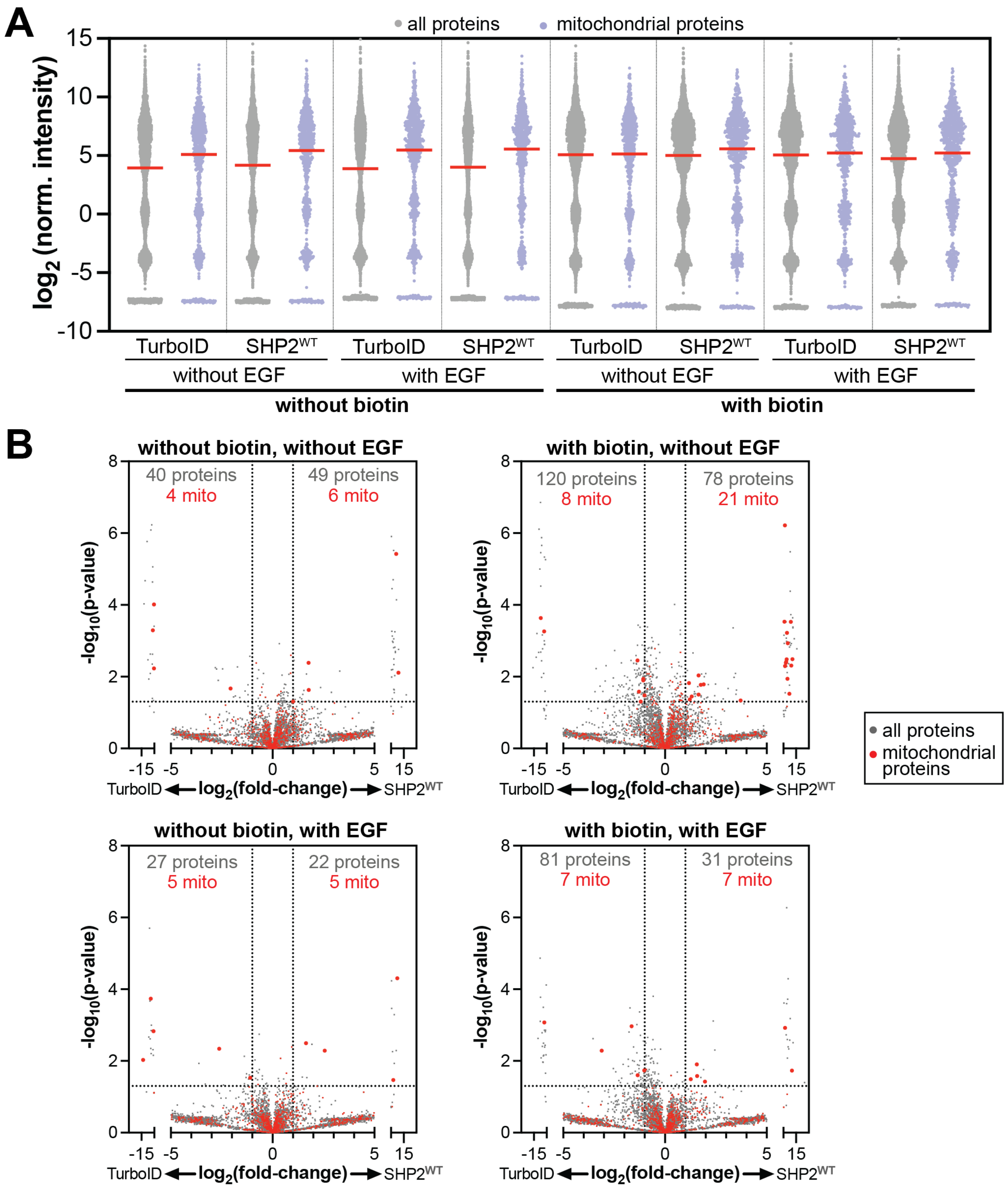

Appendix Figure S3. Effects of biotin on mitochondrial TurboID signal. (**A**) Comparison of the distributions of normalized intensity in the TurboID mass spectrometry data across the whole proteome, or the sub-proteome annotated to have mitochondrial localization, in samples treated with or without EGF, and with or without exogenous biotin. The red bars denote the median of the distribution (n = 3 biological replicates for each condition). (**B**) Volcano plots comparing SHP2^{WT}-TurboID to the TurboID-only control, in samples treated with or without EGF, and with or without exogenous biotin. Counts for proteins significantly enriched on the TurboID control or SHP2^{WT}-TurboID are given within the graphs. n = 3 biological replicates for each condition. p-values were determined using a heteroscedastic, two-tailed t-test.

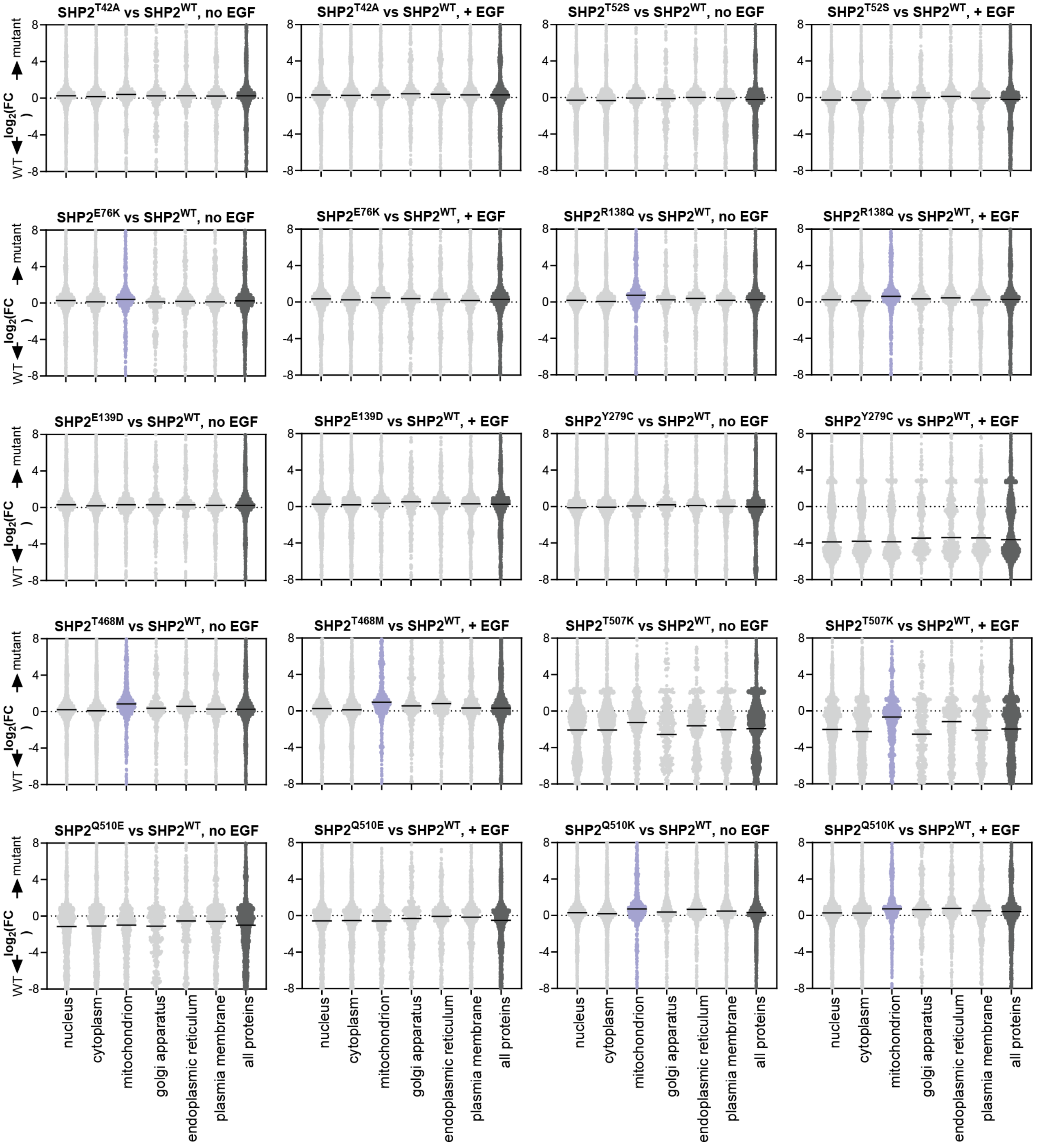

Appendix Figure S4. SHP2 proximity-labeling distributions in subcellular compartments. Each graph shows a series of distributions made up of proteins found in different subcellular compartments. The distributions show the enrichment values for those proteins in SHP2-TurboID samples relative to the TurboID control (n = 3 biological replicates for both groups). Subcellular distributions with a distinctive increase relative to the "all proteins" distribution (dark gray) are highlighted in color.

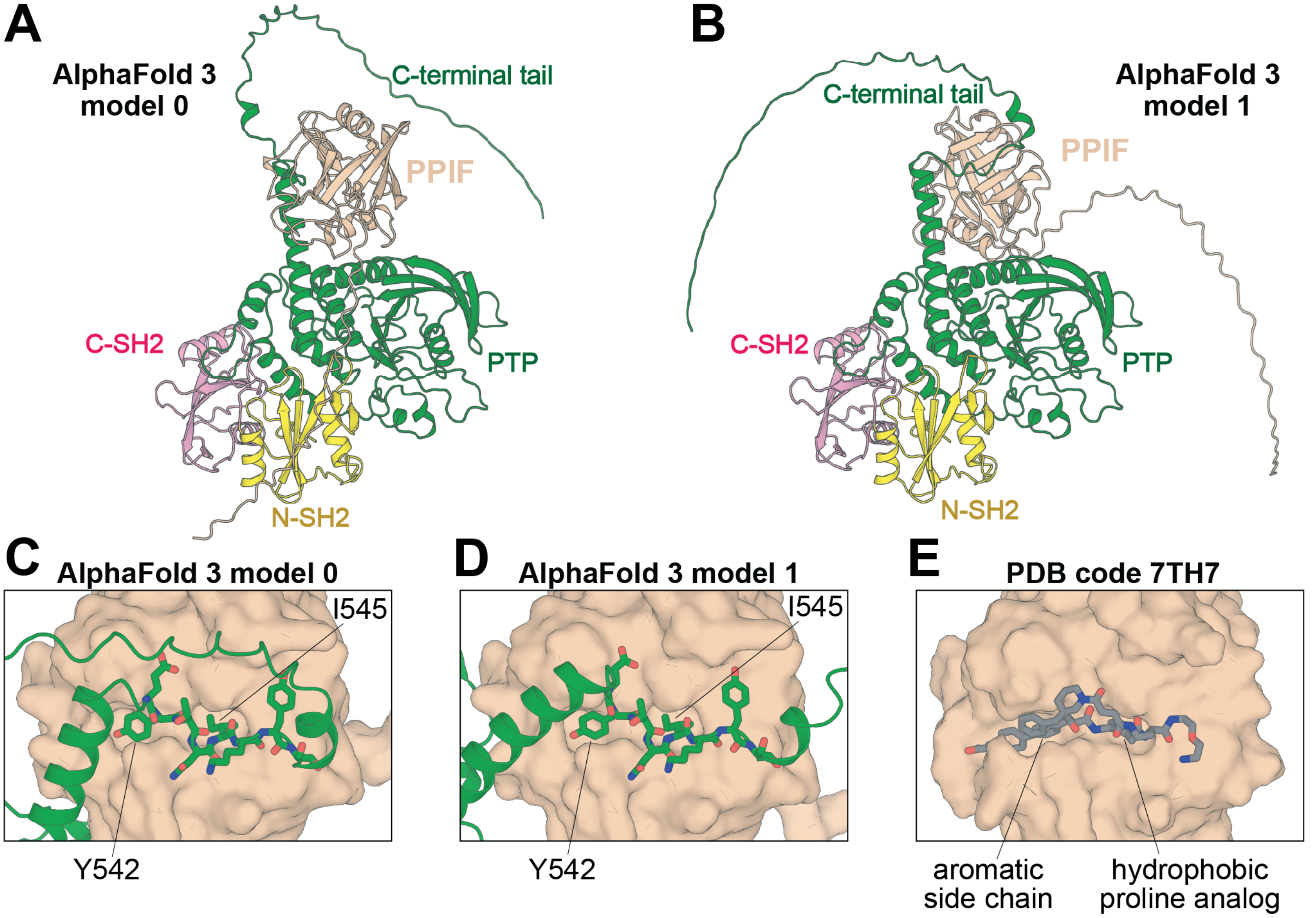

Appendix Figure S5. AlphaFold 3 models of the SHP2-PPIF interaction. (**A**) Top AlphaFold 3 model of full-length human SHP2 and PPIF proteins. ipTM score = 0.51. (**B**) Second best AlphaFold 3 model of full-length human SHP2 and PPIF proteins. ipTM score = 0.51. (**C**) and (**D**) Zoomed-in views of the models in panels (A) and (B), showing residues 541-548 in sticks docked into the active site of PPIF. (**E**) Crystal structure of PPIF bound to a macrocyclic inhibitor (PDB code 7TH7), with key aromatic and hydrophobic side chains making similar contacts to those seen in the SHP2-PPIF AlphaFold 3 models.
