# Supplementary figures and images for: Proximity-labeling proteomics reveals remodeled interactomes and altered localization of pathogenic SHP2 variants
Source: EMBO Rep. 2025 Dec 22;27(3):793–826. doi: 10.1038/s44319-025-00674-4 (PMC12894930; doi:10.1038/s44319-025-00674-4)

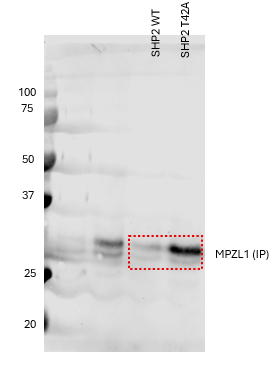

Supplement: Supplementary file 11 — Source data Fig. 4 [file 44319_2025_674_MOESM11_ESM.zip › Figure 4/D/Western_MPZL1 (IP).png]

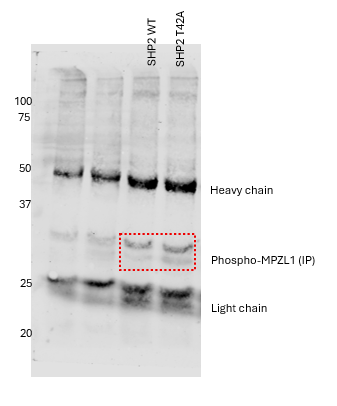

Supplement: Supplementary file 11 — Source data Fig. 4 [file 44319_2025_674_MOESM11_ESM.zip › Figure 4/D/Western_pTyr (IP).png]

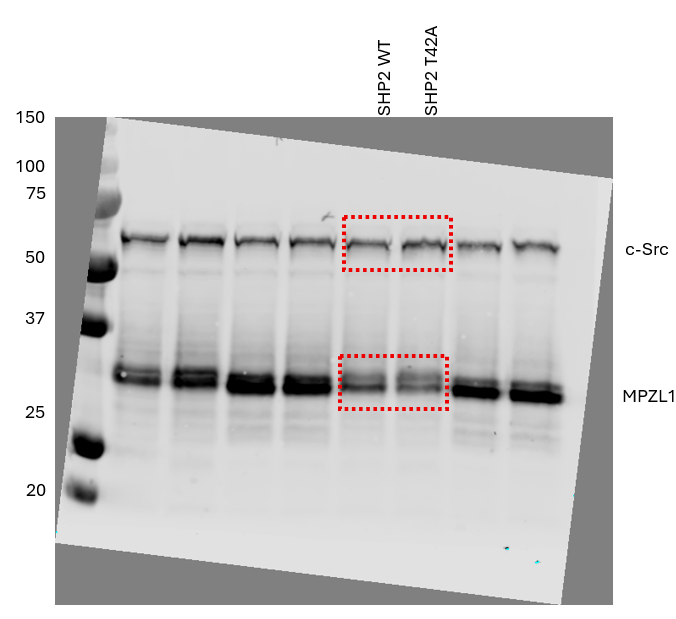

Supplement: Supplementary file 11 — Source data Fig. 4 [file 44319_2025_674_MOESM11_ESM.zip › Figure 4/D/Western_c-Src, MPZL1(lysate).png]

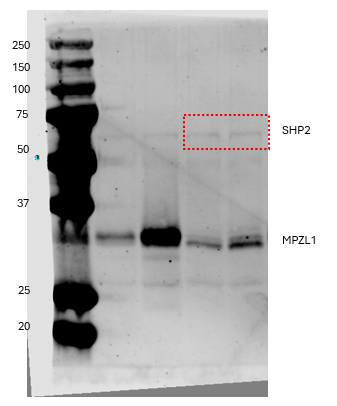

Supplement: Supplementary file 11 — Source data Fig. 4 [file 44319_2025_674_MOESM11_ESM.zip › Figure 4/D/Western_SHP2, MPZL1 (IP).png]

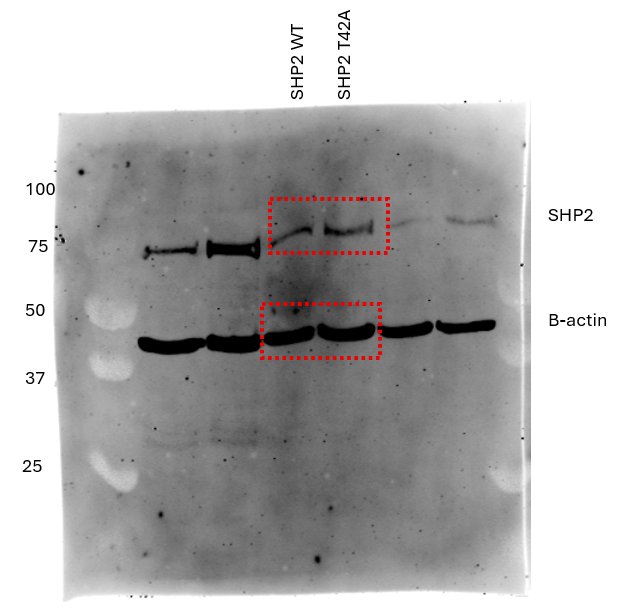

Supplement: Supplementary file 11 — Source data Fig. 4 [file 44319_2025_674_MOESM11_ESM.zip › Figure 4/D/Western_SHP2, B-actin (lysate).png]

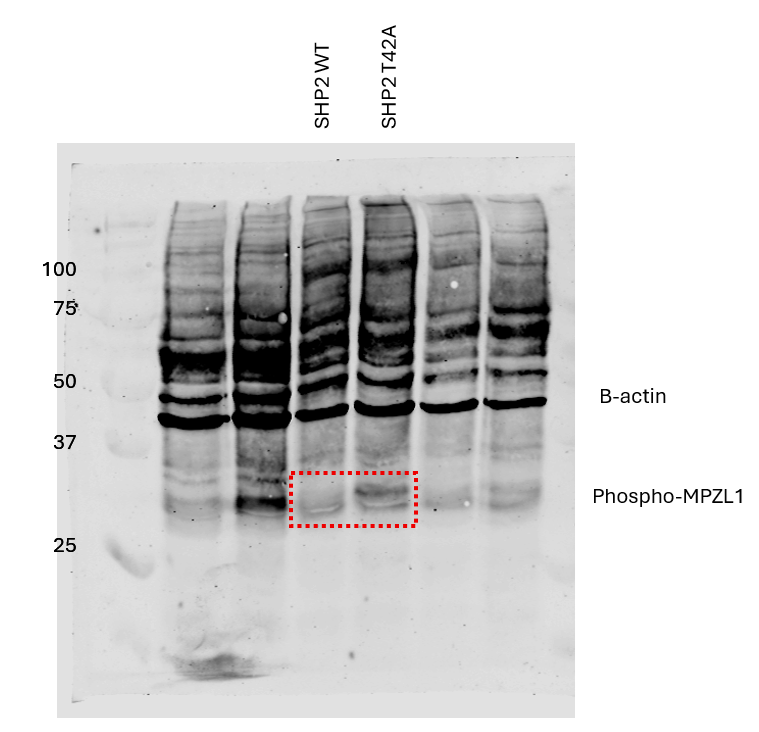

Supplement: Supplementary file 11 — Source data Fig. 4 [file 44319_2025_674_MOESM11_ESM.zip › Figure 4/D/Western_pTyr, B-actin (lysate).png]

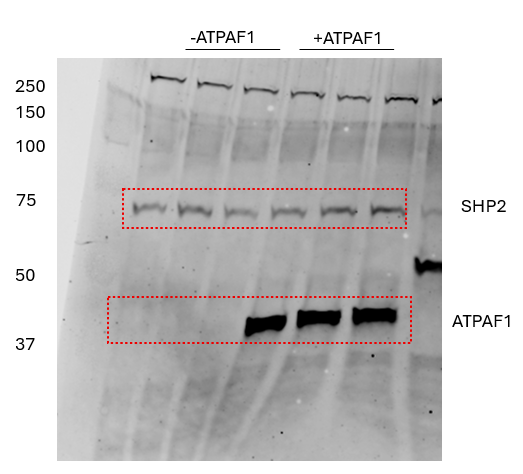

Supplement: Supplementary file 12 — Source data Fig. 5 [file 44319_2025_674_MOESM12_ESM.zip › Figure 5/G/Western_SHP2, ATPAF1 (lysate).png]

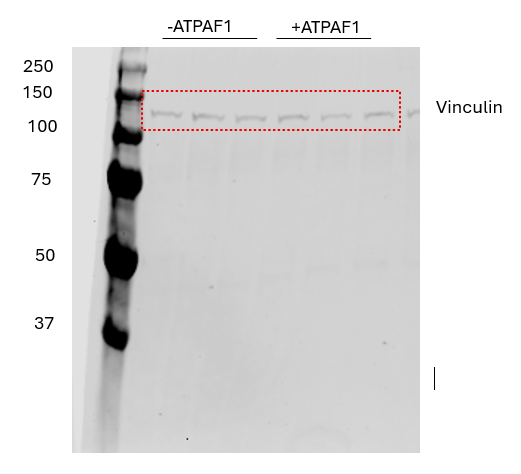

Supplement: Supplementary file 12 — Source data Fig. 5 [file 44319_2025_674_MOESM12_ESM.zip › Figure 5/G/Western_Vinculin (lysate).png]

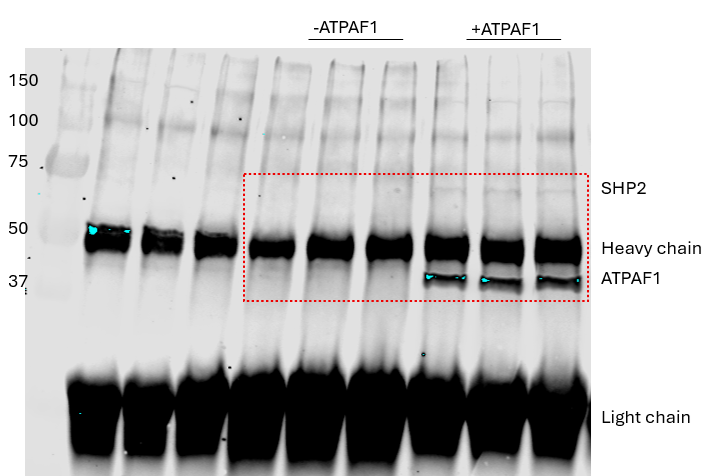

Supplement: Supplementary file 12 — Source data Fig. 5 [file 44319_2025_674_MOESM12_ESM.zip › Figure 5/G/Western_SHP2, ATPAF1 (IP).png]

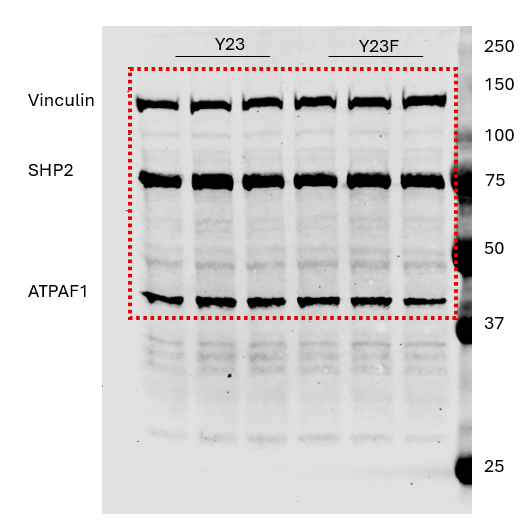

Supplement: Supplementary file 12 — Source data Fig. 5 [file 44319_2025_674_MOESM12_ESM.zip › Figure 5/H/Western_SHP2, ATPAF1, Vinculin (lysate).png]

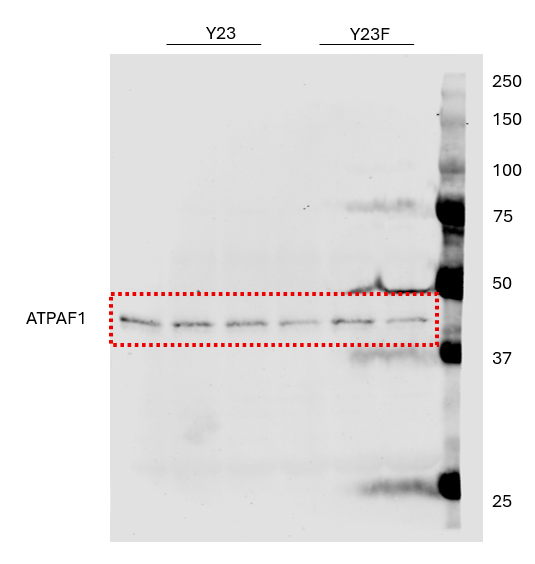

Supplement: Supplementary file 12 — Source data Fig. 5 [file 44319_2025_674_MOESM12_ESM.zip › Figure 5/H/Western_ATPAF1 (IP).png]

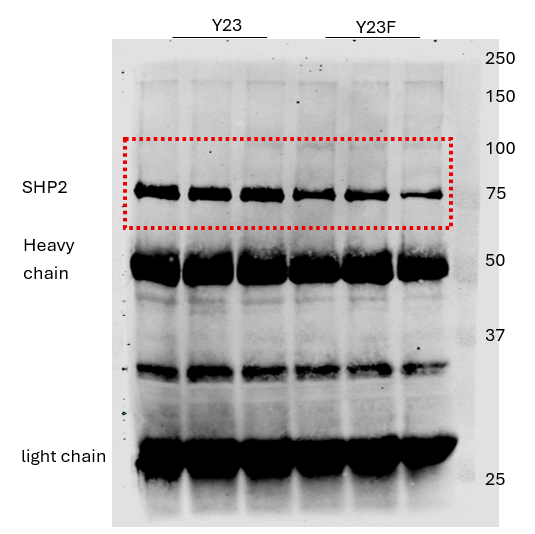

Supplement: Supplementary file 12 — Source data Fig. 5 [file 44319_2025_674_MOESM12_ESM.zip › Figure 5/H/Western_SHP2 (IP).png]

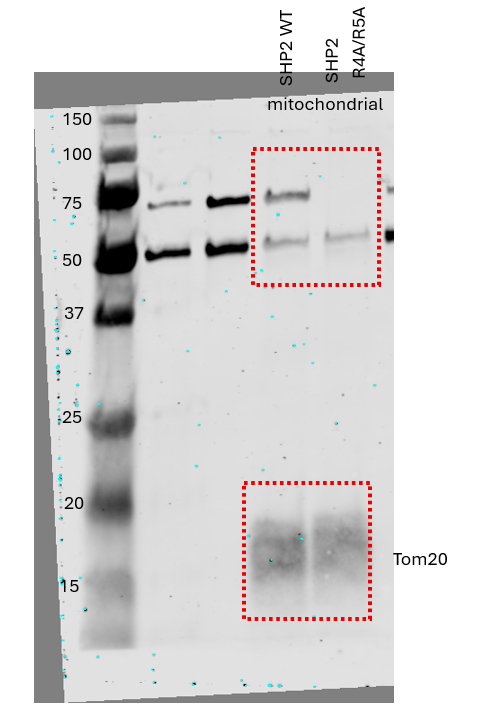

Supplement: Supplementary file 12 — Source data Fig. 5 [file 44319_2025_674_MOESM12_ESM.zip › Figure 5/C/Western_SHP2, A-Tubulin, Tom20_ SHP2 WT, R4A R5A (mitochondrial).png]

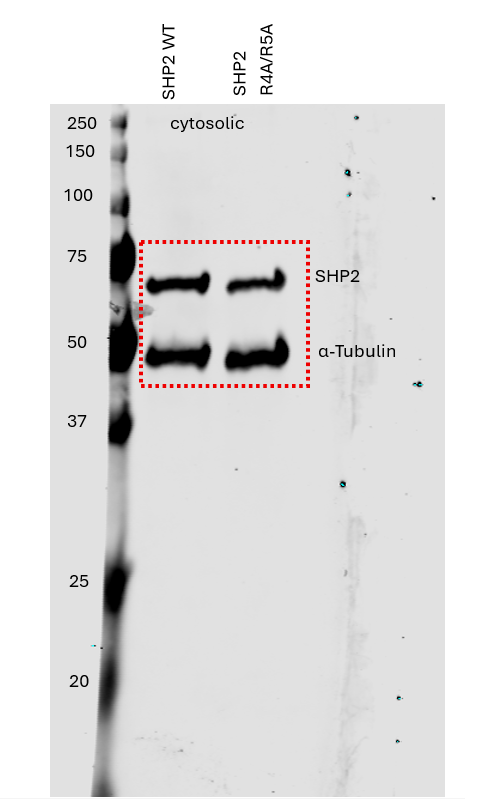

Supplement: Supplementary file 12 — Source data Fig. 5 [file 44319_2025_674_MOESM12_ESM.zip › Figure 5/C/Western_SHP2, A-Tubulin_ SHP2 WT, R4A R5A (cytosolic).png]

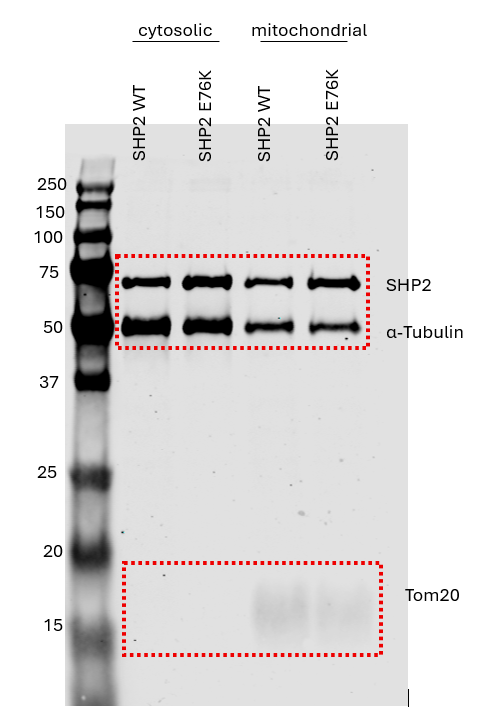

Supplement: Supplementary file 12 — Source data Fig. 5 [file 44319_2025_674_MOESM12_ESM.zip › Figure 5/C/Western_SHP2, A-Tubulin, Tom20_ SHP2 WT, E76K.png]

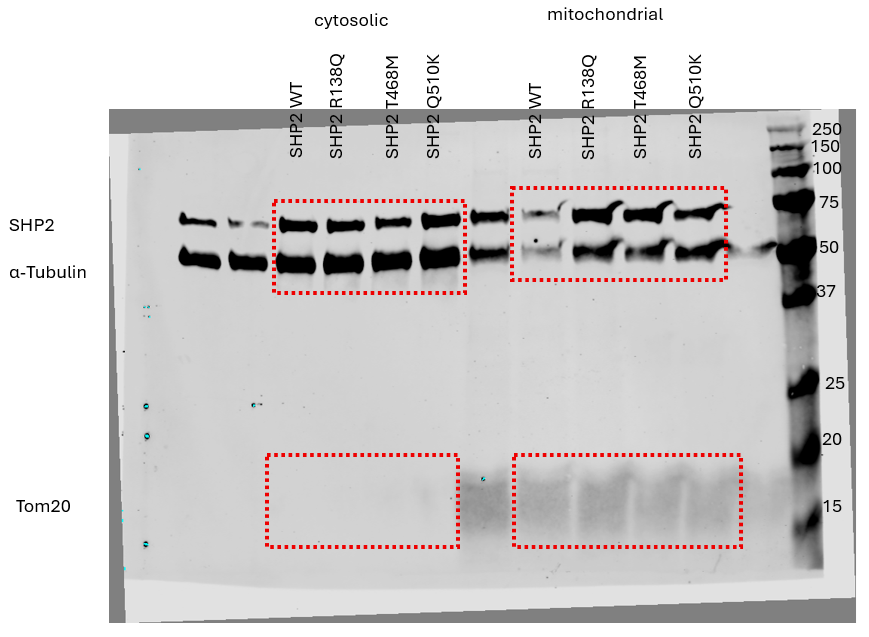

Supplement: Supplementary file 12 — Source data Fig. 5 [file 44319_2025_674_MOESM12_ESM.zip › Figure 5/C/Western_SHP2, A-Tubulin, Tom20_ SHP2 WT, R138Q, T468M, Q510K.png]

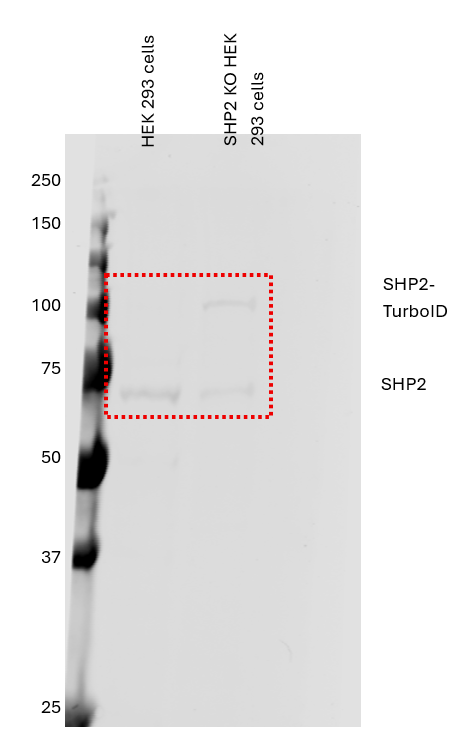

Supplement: Supplementary file 15 — Figure EV1 Source Data [file 44319_2025_674_MOESM15_ESM.zip › Figure EV1/F/Western_SHP2.png]

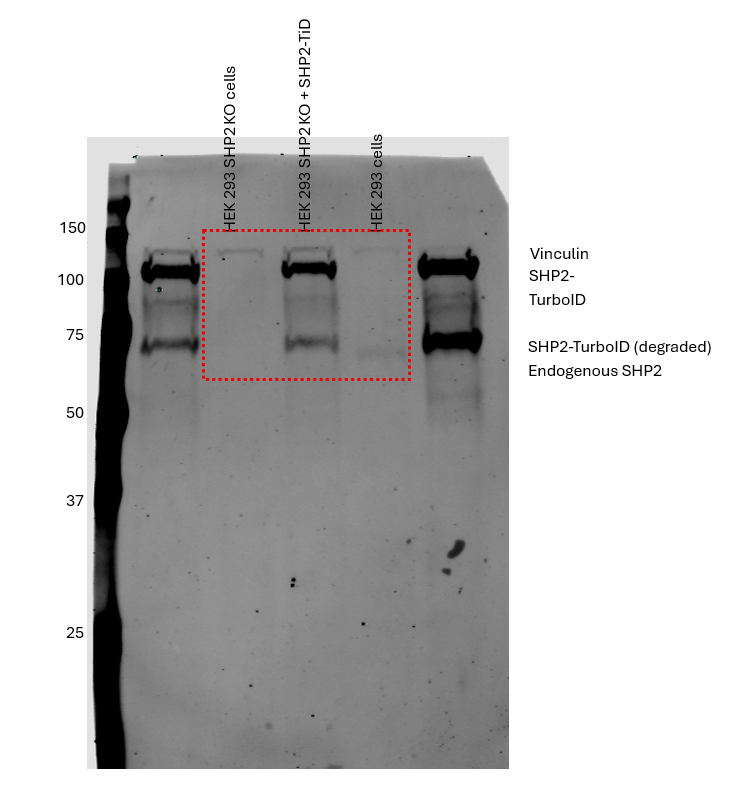

Supplement: Supplementary file 15 — Figure EV1 Source Data [file 44319_2025_674_MOESM15_ESM.zip › Figure EV1/F/Western_Vinculin_SHP2.png]

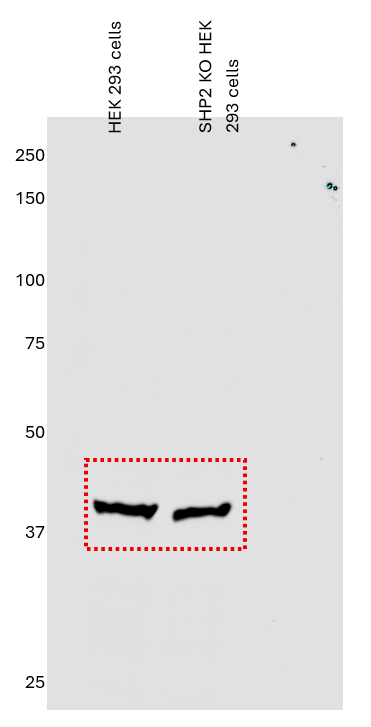

Supplement: Supplementary file 15 — Figure EV1 Source Data [file 44319_2025_674_MOESM15_ESM.zip › Figure EV1/F/Western_B-actin.png]

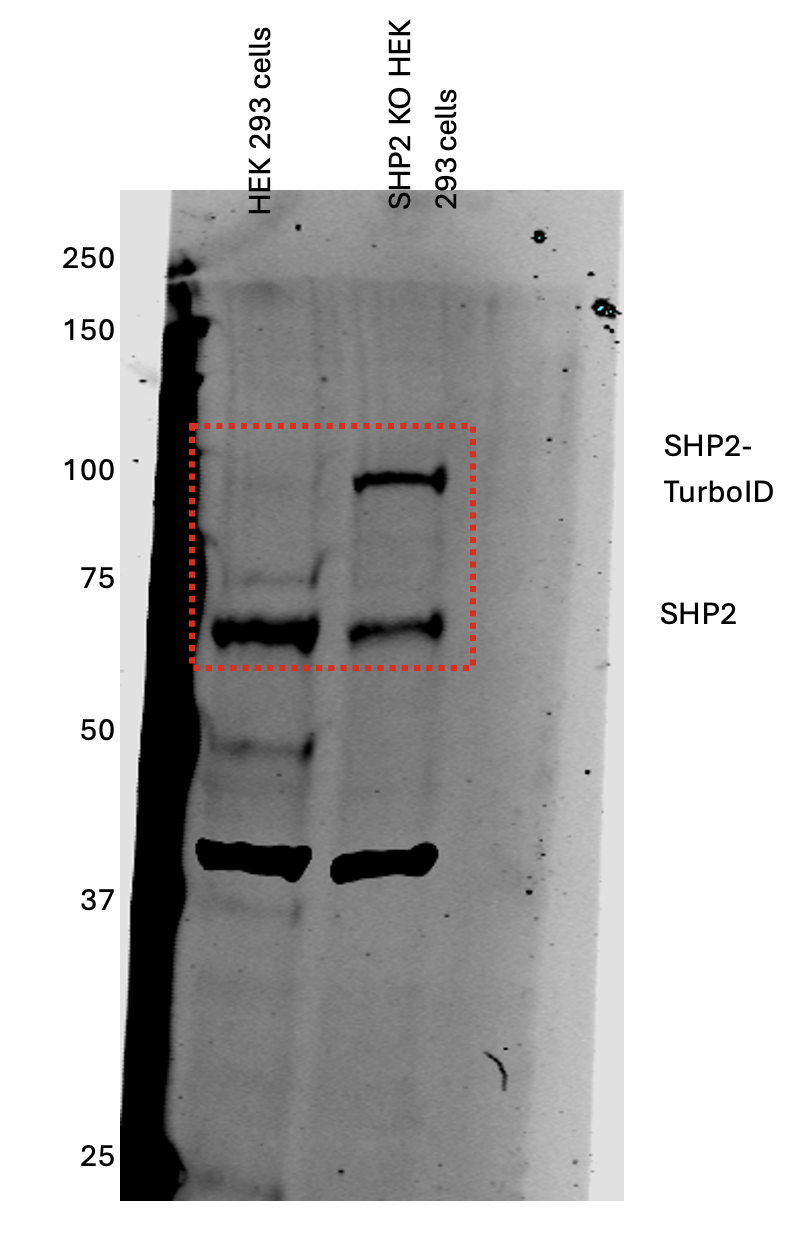

Supplement: Supplementary file 15 — Figure EV1 Source Data [file 44319_2025_674_MOESM15_ESM.zip › Figure EV1/F/Western_SHP2-actin-darker.png]

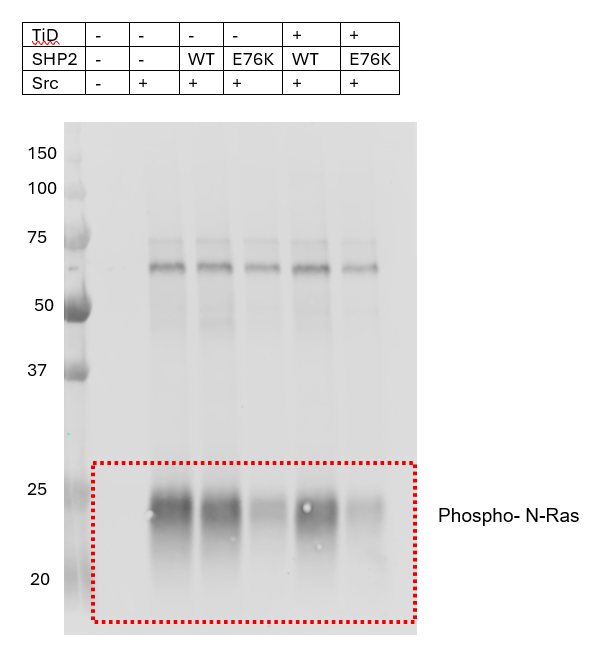

Supplement: Supplementary file 15 — Figure EV1 Source Data [file 44319_2025_674_MOESM15_ESM.zip › Figure EV1/D/Western_pTyr (IP).png]

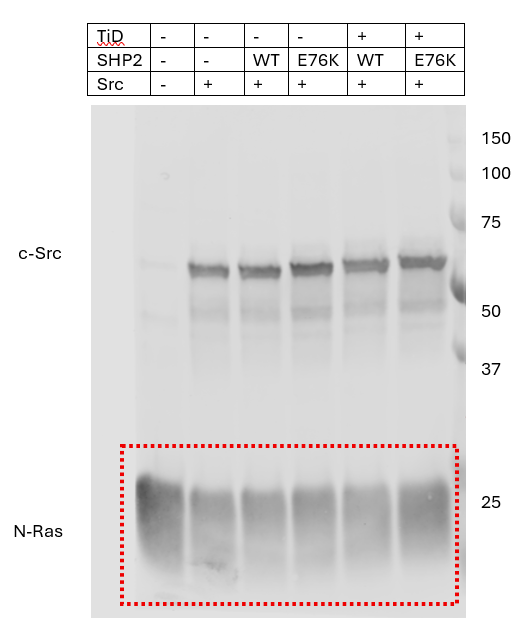

Supplement: Supplementary file 15 — Figure EV1 Source Data [file 44319_2025_674_MOESM15_ESM.zip › Figure EV1/D/Western_c-Src, N-Ras (lysate).png]

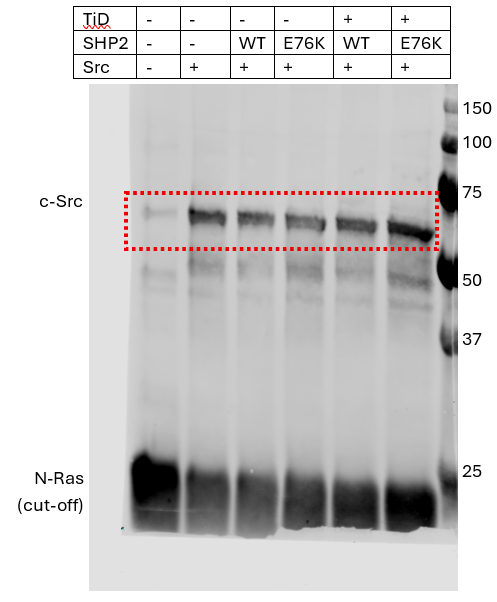

Supplement: Supplementary file 15 — Figure EV1 Source Data [file 44319_2025_674_MOESM15_ESM.zip › Figure EV1/D/Western_c-Src (lysate).png]

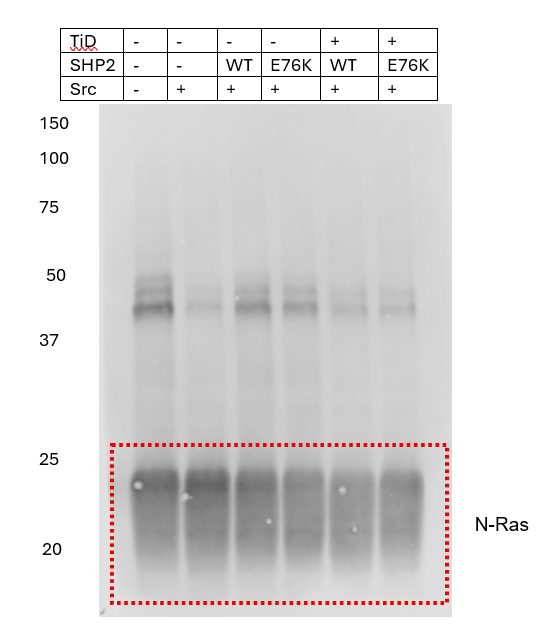

Supplement: Supplementary file 15 — Figure EV1 Source Data [file 44319_2025_674_MOESM15_ESM.zip › Figure EV1/D/Western_N-Ras (IP).png]

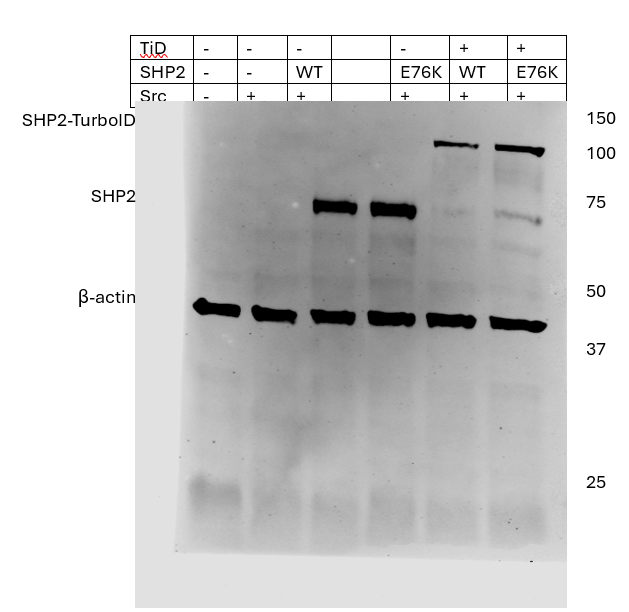

Supplement: Supplementary file 15 — Figure EV1 Source Data [file 44319_2025_674_MOESM15_ESM.zip › Figure EV1/D/Western_SHP2, B-Actin (lysate).png]

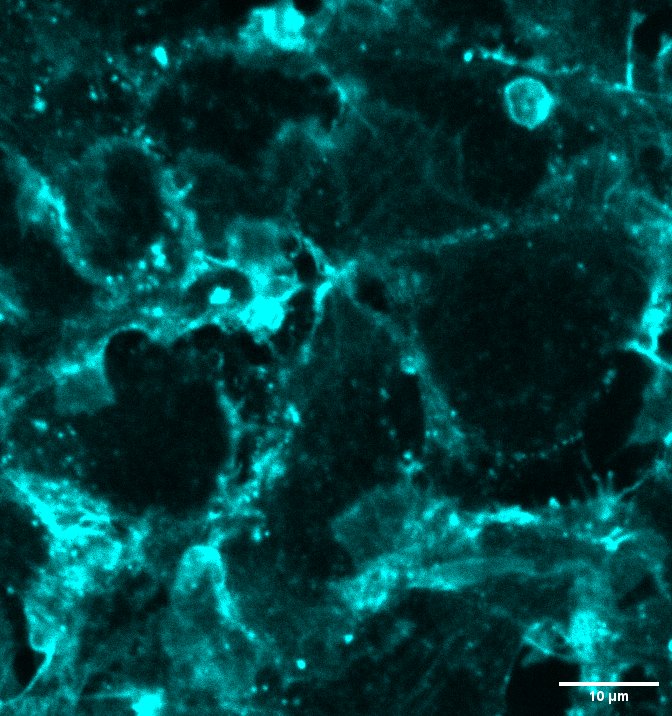

Supplement: Supplementary file 18 — Figure EV4 Source Data [file 44319_2025_674_MOESM18_ESM.zip › Figure EV4/A/D1 (endogenous, stimulated)/D1_F002_Z9_Phalloidin.jpg]

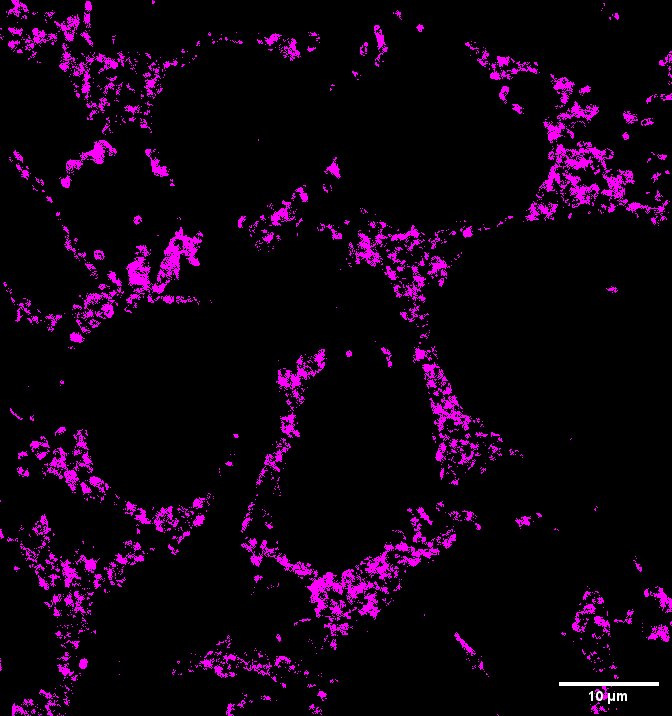

Supplement: Supplementary file 18 — Figure EV4 Source Data [file 44319_2025_674_MOESM18_ESM.zip › Figure EV4/A/D1 (endogenous, stimulated)/D1_F002_Z9_Overlap_SHP2_Mito.jpg]

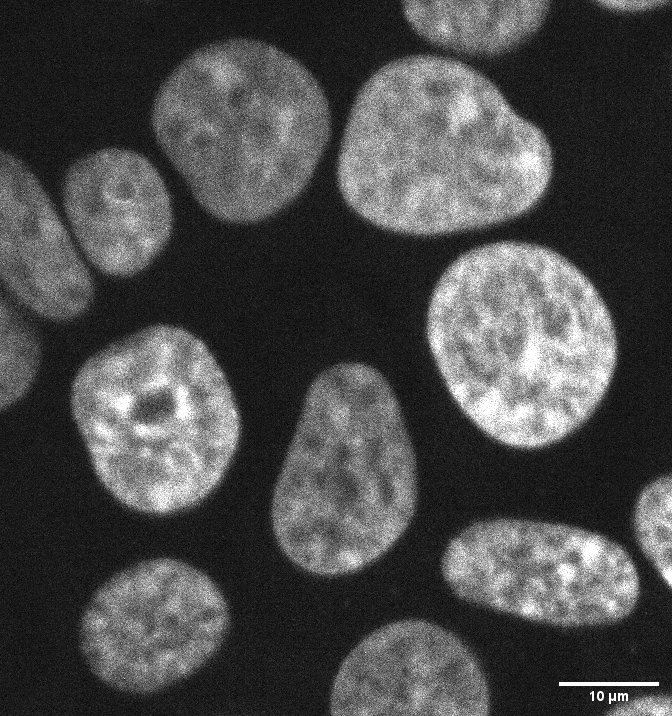

Supplement: Supplementary file 18 — Figure EV4 Source Data [file 44319_2025_674_MOESM18_ESM.zip › Figure EV4/A/D1 (endogenous, stimulated)/D1_F002_Z9_DAPI.jpg]

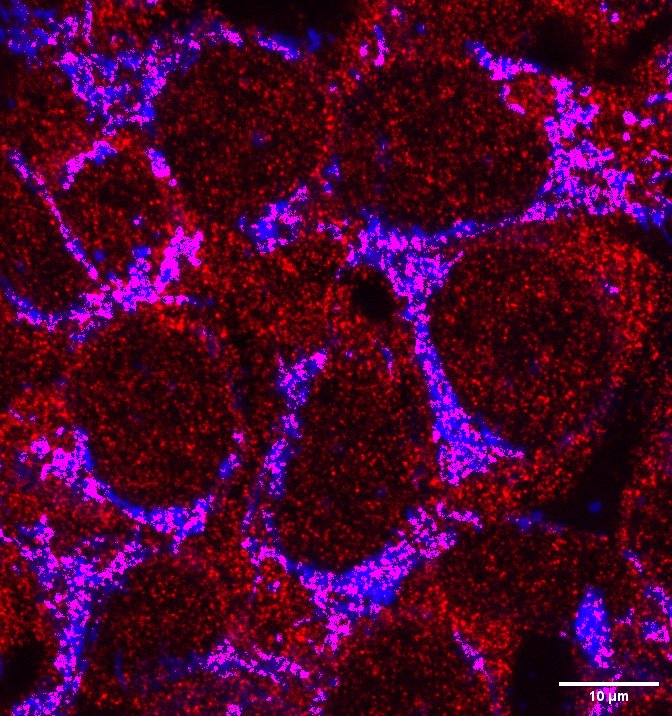

Supplement: Supplementary file 18 — Figure EV4 Source Data [file 44319_2025_674_MOESM18_ESM.zip › Figure EV4/A/D1 (endogenous, stimulated)/D1_F002_Z9_Overlay_SHP2_Mito.jpg]

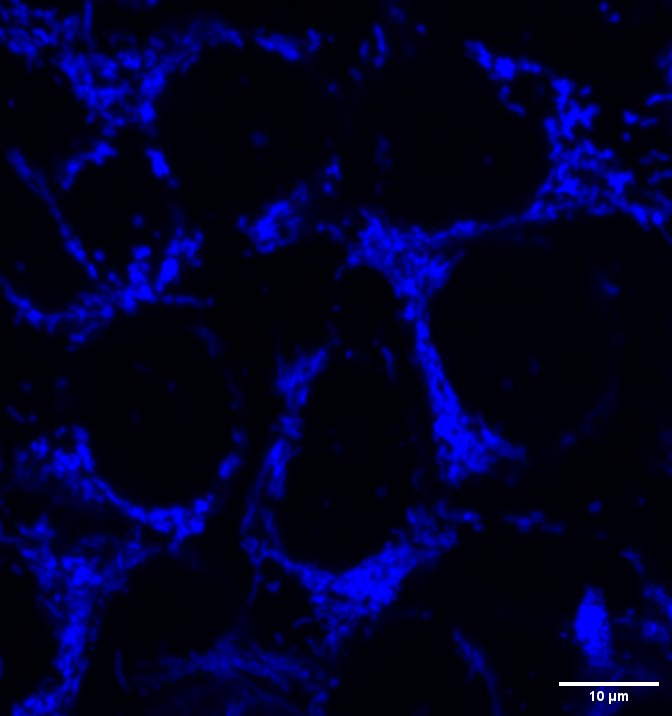

Supplement: Supplementary file 18 — Figure EV4 Source Data [file 44319_2025_674_MOESM18_ESM.zip › Figure EV4/A/D1 (endogenous, stimulated)/D1_F002_Z9_mito.jpg]

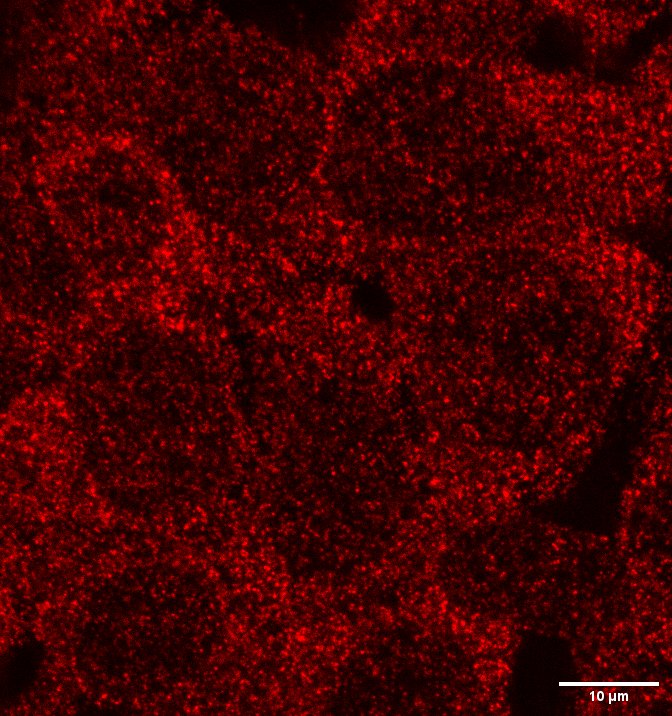

Supplement: Supplementary file 18 — Figure EV4 Source Data [file 44319_2025_674_MOESM18_ESM.zip › Figure EV4/A/D1 (endogenous, stimulated)/D1_F002_Z9_SHP2.jpg]

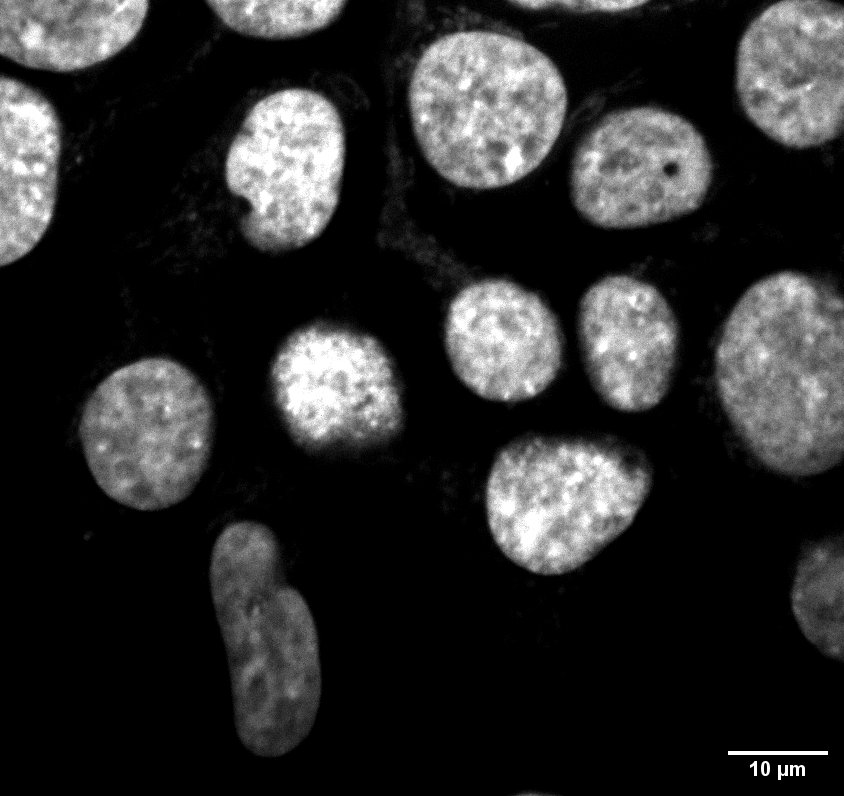

Supplement: Supplementary file 18 — Figure EV4 Source Data [file 44319_2025_674_MOESM18_ESM.zip › Figure EV4/A/C1 (endogenous, unstimulated)/C1_F001_Z9_DAPI.jpg]

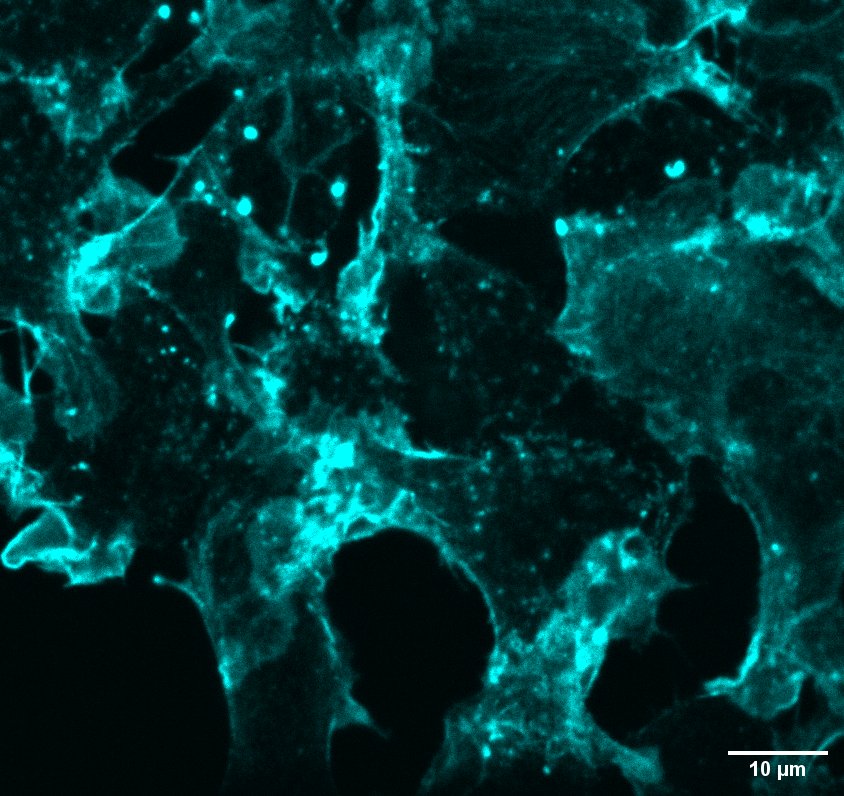

Supplement: Supplementary file 18 — Figure EV4 Source Data [file 44319_2025_674_MOESM18_ESM.zip › Figure EV4/A/C1 (endogenous, unstimulated)/C1_F001_Z9_Phalloidin.jpg]

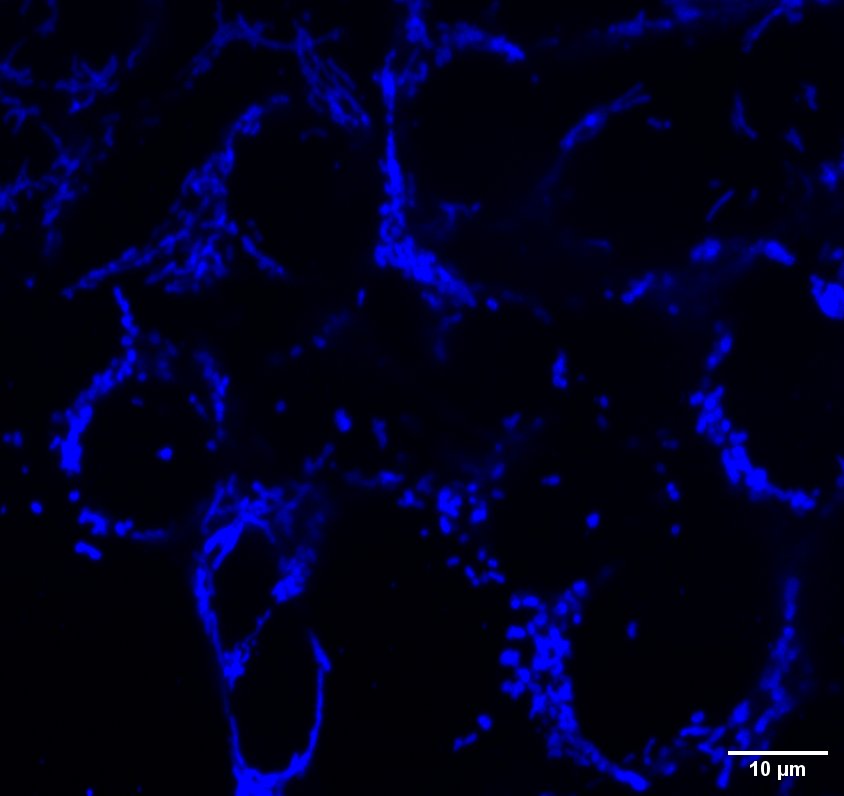

Supplement: Supplementary file 18 — Figure EV4 Source Data [file 44319_2025_674_MOESM18_ESM.zip › Figure EV4/A/C1 (endogenous, unstimulated)/C1_F001_Z9_mito.jpg]

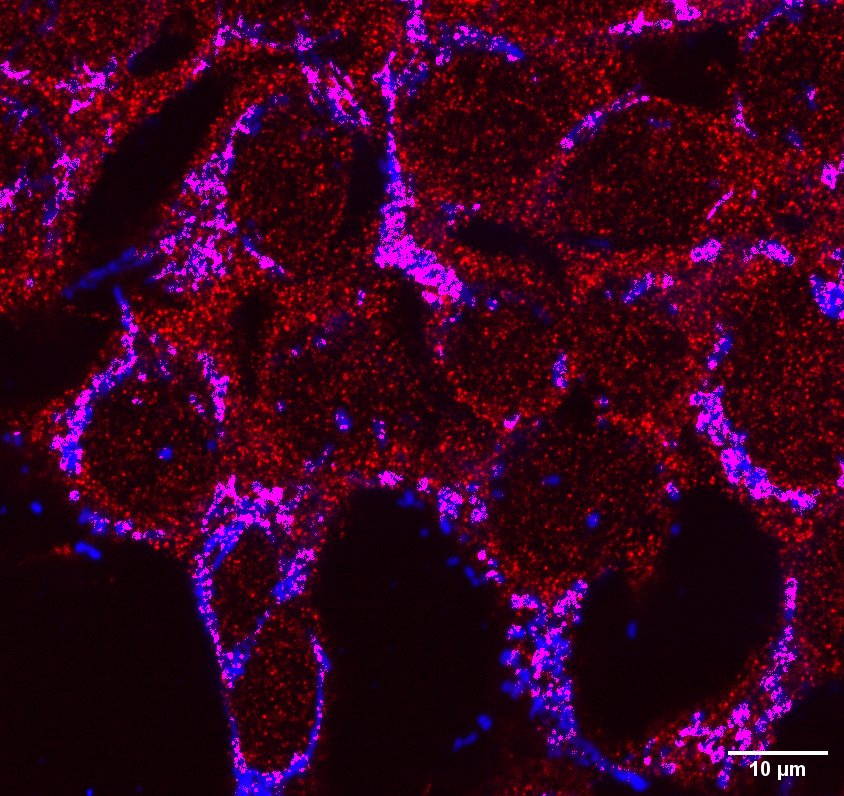

Supplement: Supplementary file 18 — Figure EV4 Source Data [file 44319_2025_674_MOESM18_ESM.zip › Figure EV4/A/C1 (endogenous, unstimulated)/C1_F001_Z9_OVERLAY_SHP2_mito.jpg]

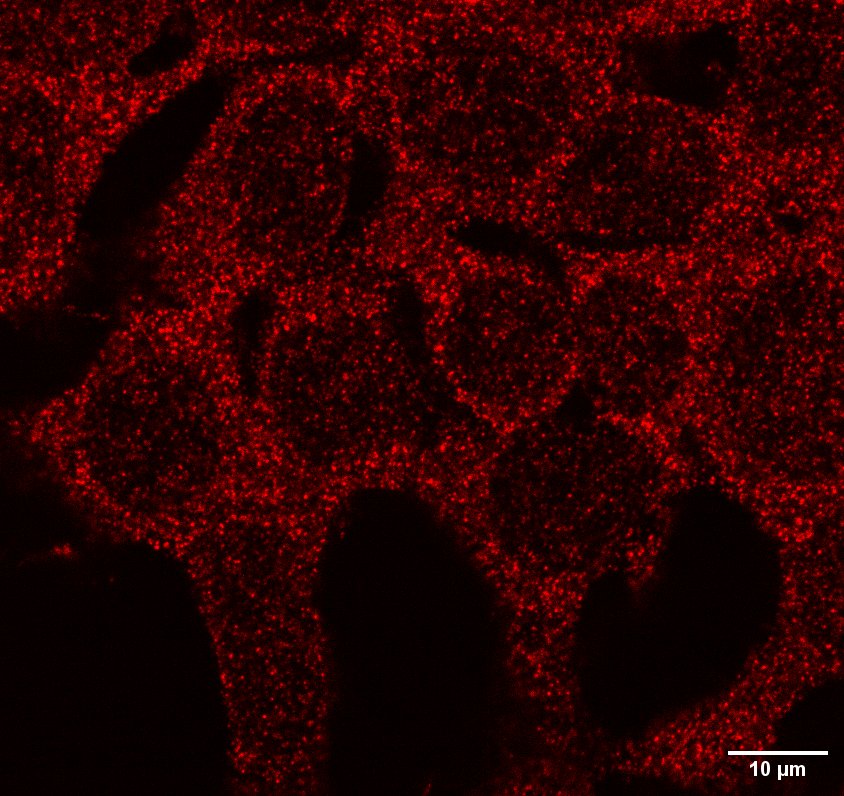

Supplement: Supplementary file 18 — Figure EV4 Source Data [file 44319_2025_674_MOESM18_ESM.zip › Figure EV4/A/C1 (endogenous, unstimulated)/C1_F001_Z9_SHP2.jpg]

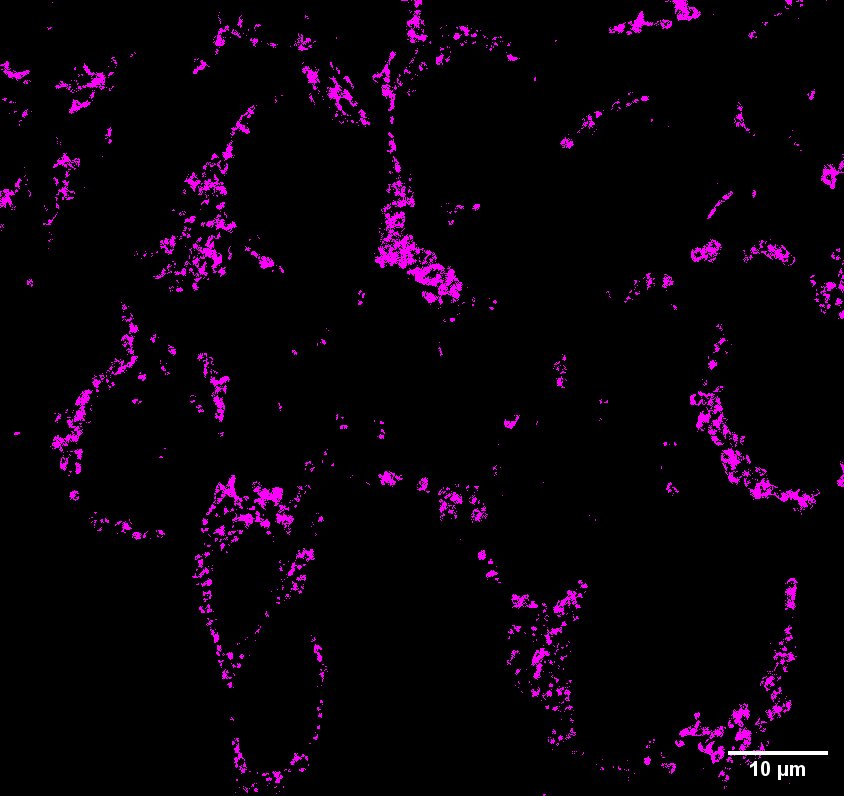

Supplement: Supplementary file 18 — Figure EV4 Source Data [file 44319_2025_674_MOESM18_ESM.zip › Figure EV4/A/C1 (endogenous, unstimulated)/C1_F001_Z9_OVERLAP_SHP2_mito.jpg]

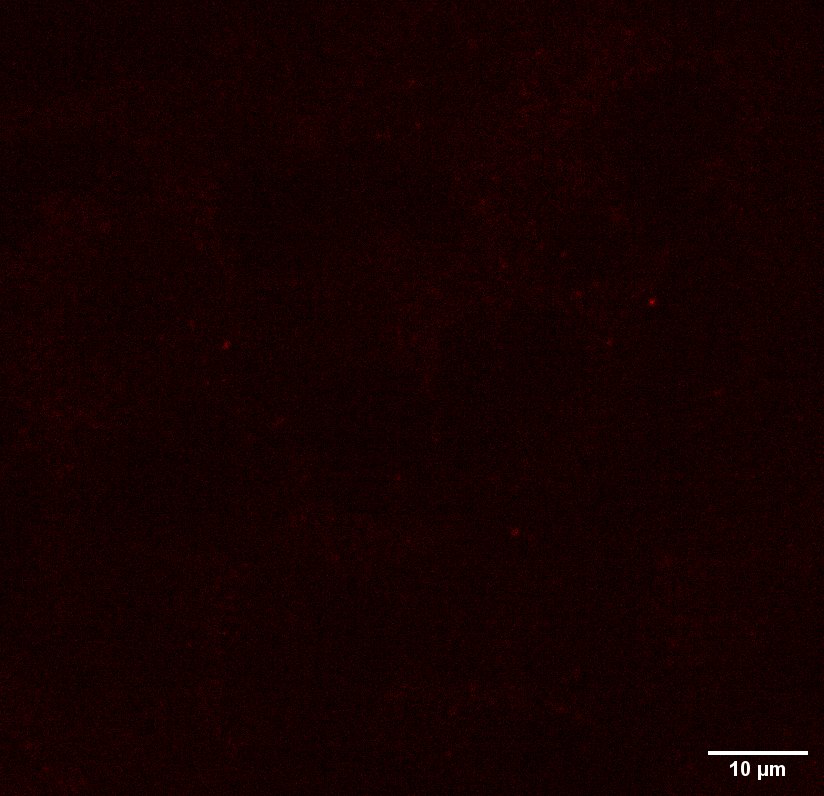

Supplement: Supplementary file 18 — Figure EV4 Source Data [file 44319_2025_674_MOESM18_ESM.zip › Figure EV4/A/B2 (SHP2 Knock-out)/B2_F004_Z9_SHP2.jpg]

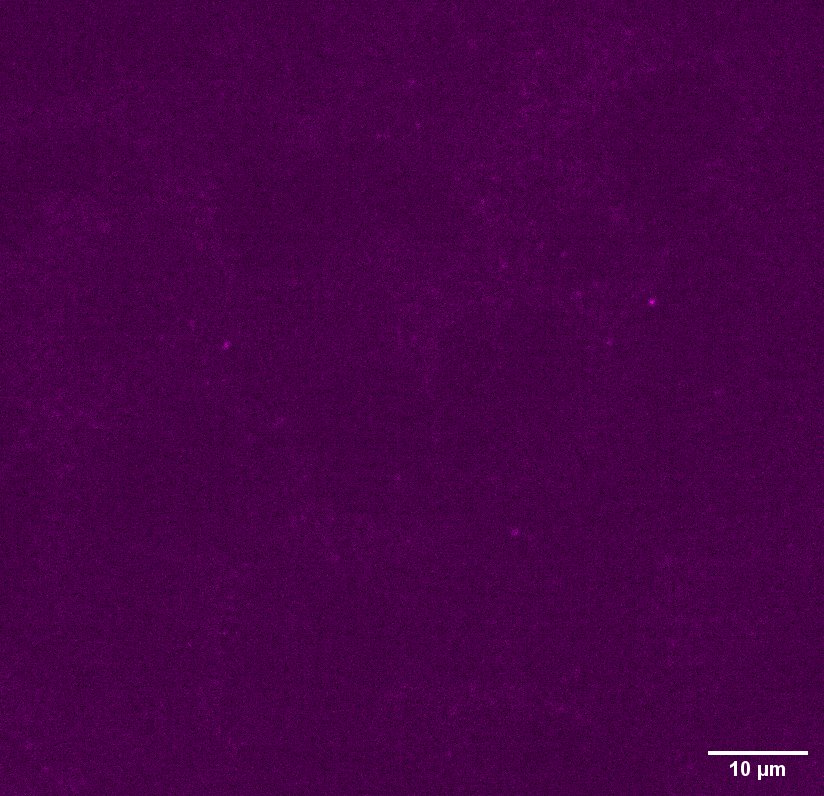

Supplement: Supplementary file 18 — Figure EV4 Source Data [file 44319_2025_674_MOESM18_ESM.zip › Figure EV4/A/B2 (SHP2 Knock-out)/B2_F004_Z9_Overlap_SHP2_mito.jpg]

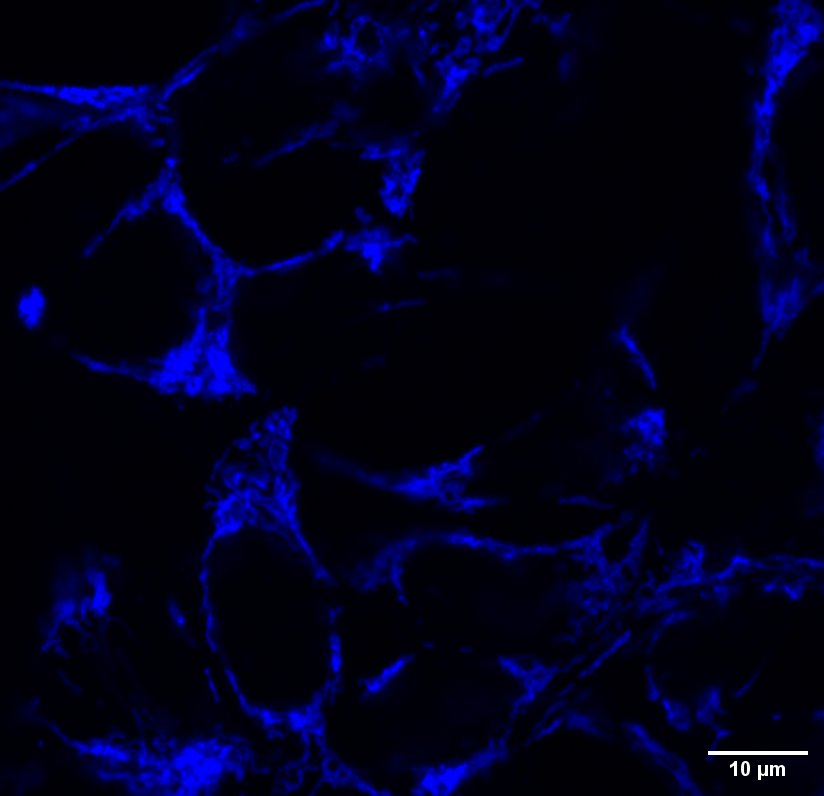

Supplement: Supplementary file 18 — Figure EV4 Source Data [file 44319_2025_674_MOESM18_ESM.zip › Figure EV4/A/B2 (SHP2 Knock-out)/B2_F004_Z9_mito.jpg]

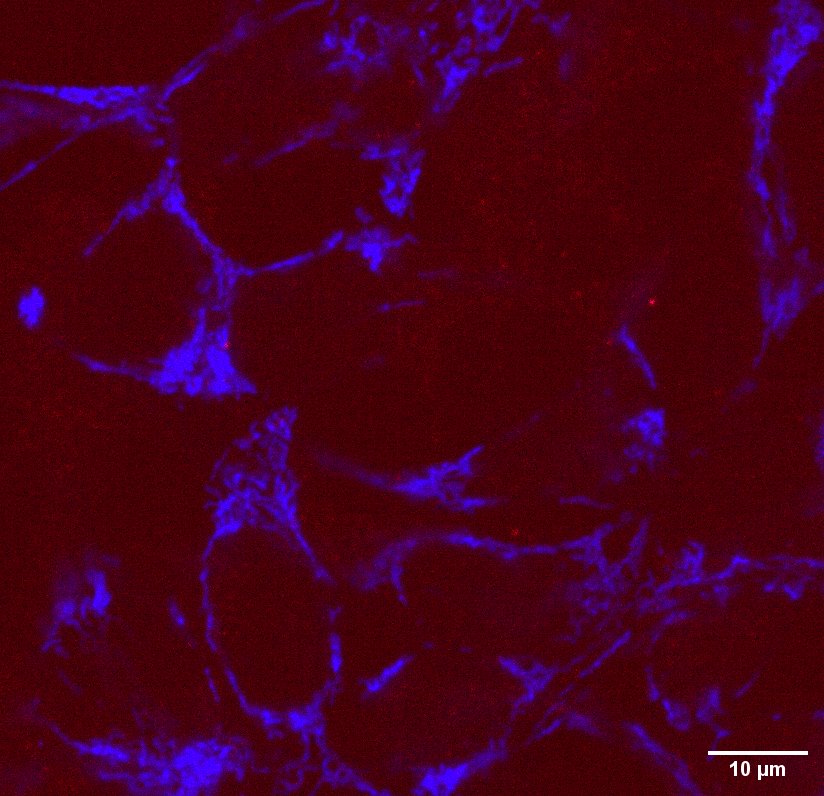

Supplement: Supplementary file 18 — Figure EV4 Source Data [file 44319_2025_674_MOESM18_ESM.zip › Figure EV4/A/B2 (SHP2 Knock-out)/B2_F004_Z9_Overlay_SHP2_mito.jpg]

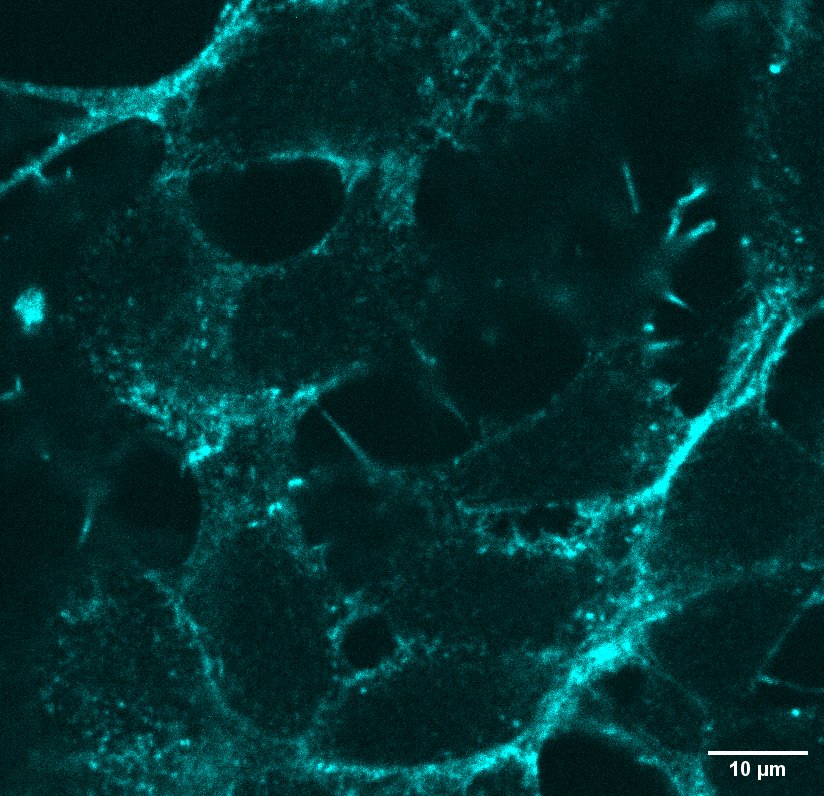

Supplement: Supplementary file 18 — Figure EV4 Source Data [file 44319_2025_674_MOESM18_ESM.zip › Figure EV4/A/B2 (SHP2 Knock-out)/B2_F004_Z9_Phalloidin.jpg]

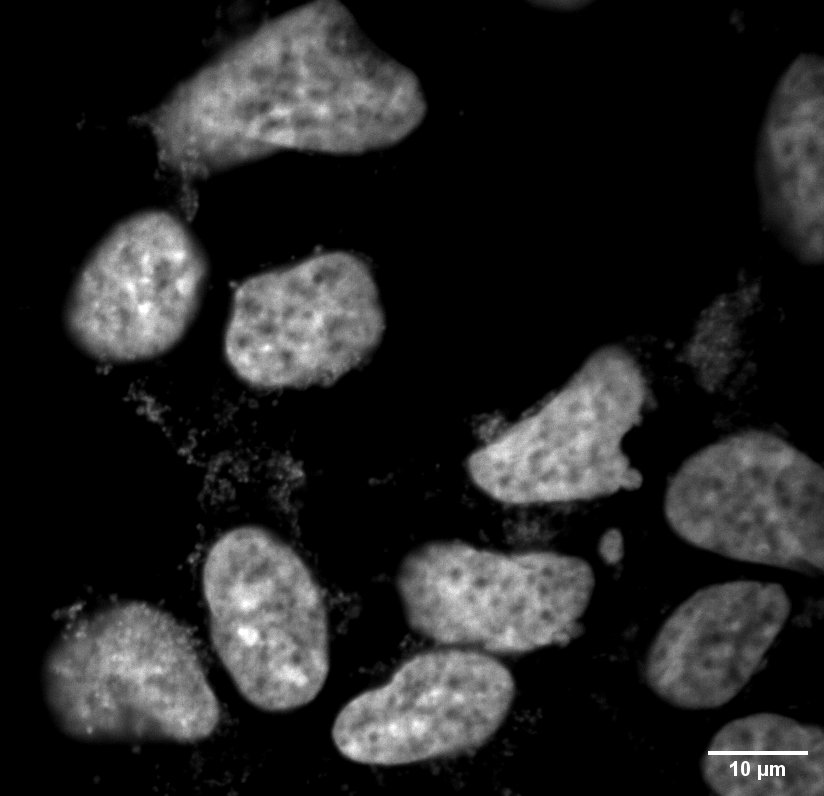

Supplement: Supplementary file 18 — Figure EV4 Source Data [file 44319_2025_674_MOESM18_ESM.zip › Figure EV4/A/B2 (SHP2 Knock-out)/B2_F004_Z9_DAPI.jpg]

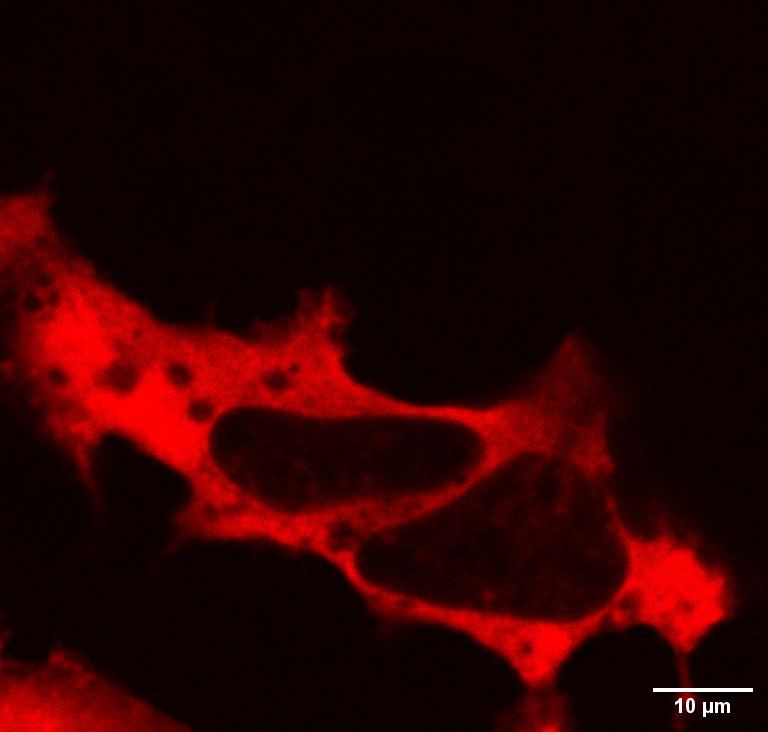

Supplement: Supplementary file 18 — Figure EV4 Source Data [file 44319_2025_674_MOESM18_ESM.zip › Figure EV4/E/B1 (SHP2-TurboID, unstimulated)/B1_F010_Z5_SHP2.jpg]

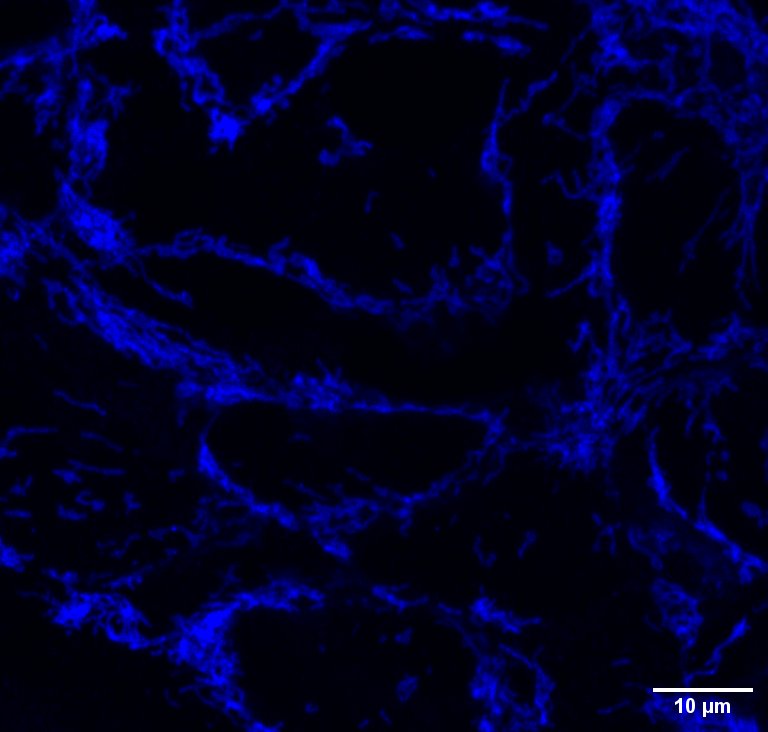

Supplement: Supplementary file 18 — Figure EV4 Source Data [file 44319_2025_674_MOESM18_ESM.zip › Figure EV4/E/B1 (SHP2-TurboID, unstimulated)/B1_F010_Z5_Mito.jpg]

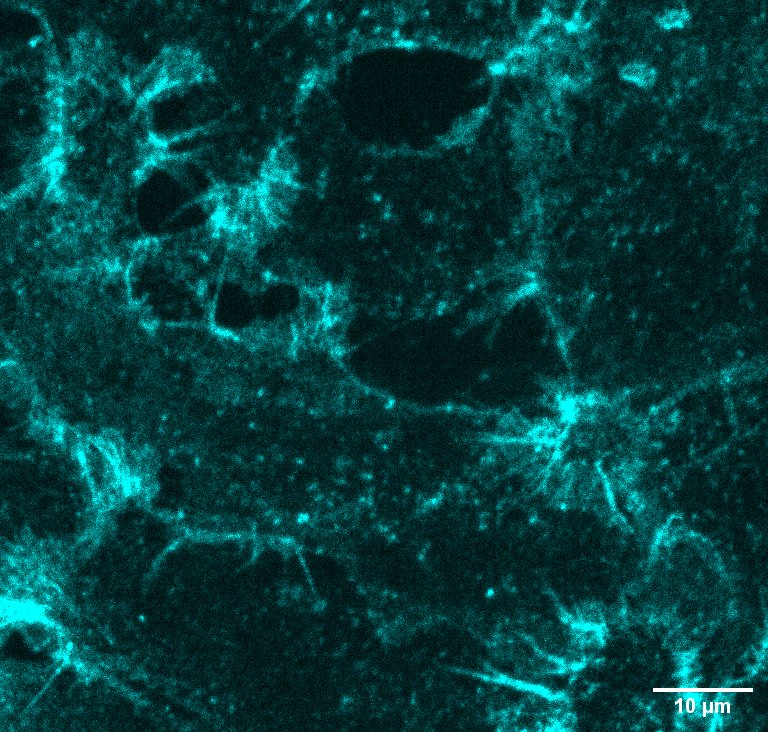

Supplement: Supplementary file 18 — Figure EV4 Source Data [file 44319_2025_674_MOESM18_ESM.zip › Figure EV4/E/B1 (SHP2-TurboID, unstimulated)/B1_F010_Z5_Phallodin.jpg]

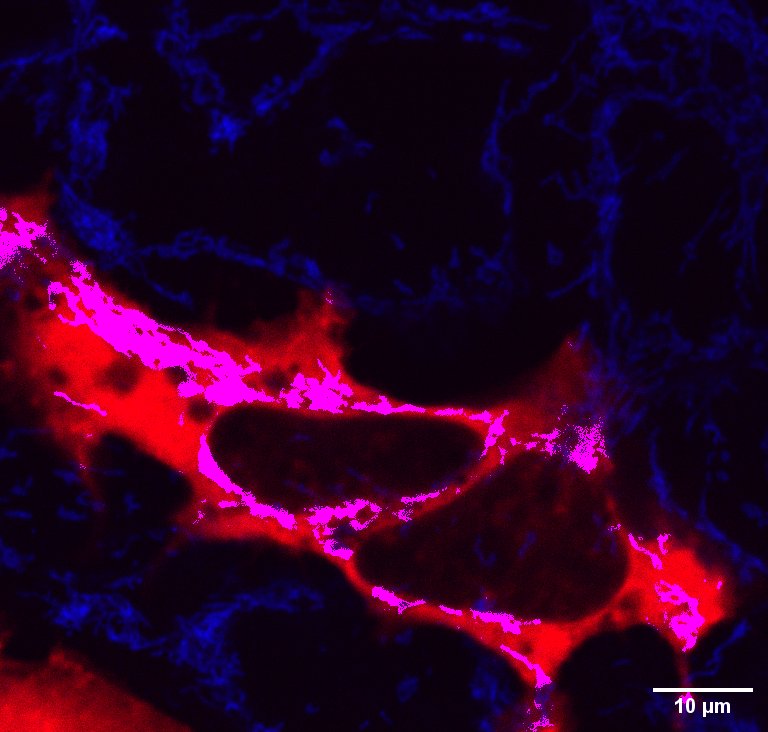

Supplement: Supplementary file 18 — Figure EV4 Source Data [file 44319_2025_674_MOESM18_ESM.zip › Figure EV4/E/B1 (SHP2-TurboID, unstimulated)/B1_F010_Z5_Overlay SHP2_Mito.jpg]

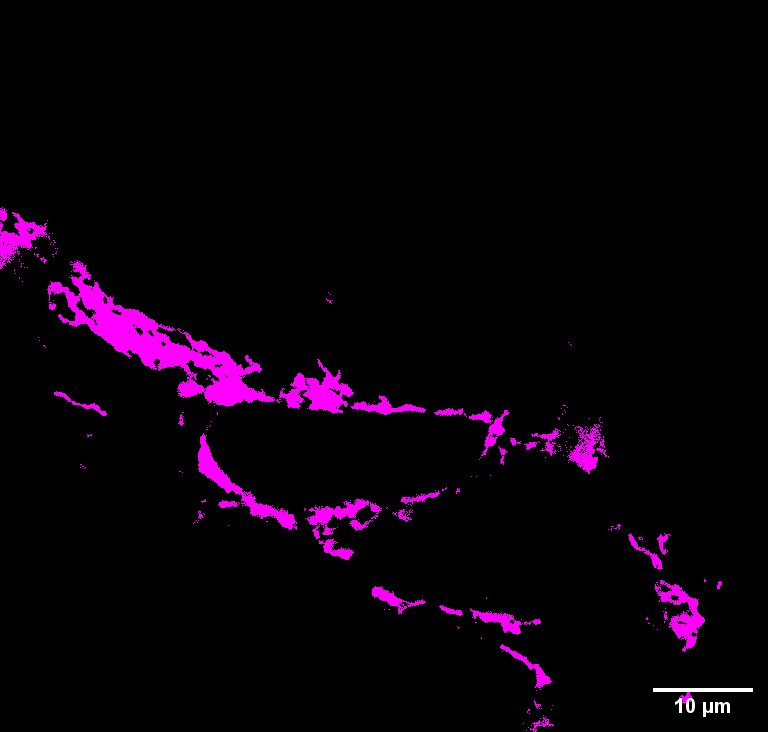

Supplement: Supplementary file 18 — Figure EV4 Source Data [file 44319_2025_674_MOESM18_ESM.zip › Figure EV4/E/B1 (SHP2-TurboID, unstimulated)/B1_F010_Z5_Overlap_SHP2_Mito.jpg]

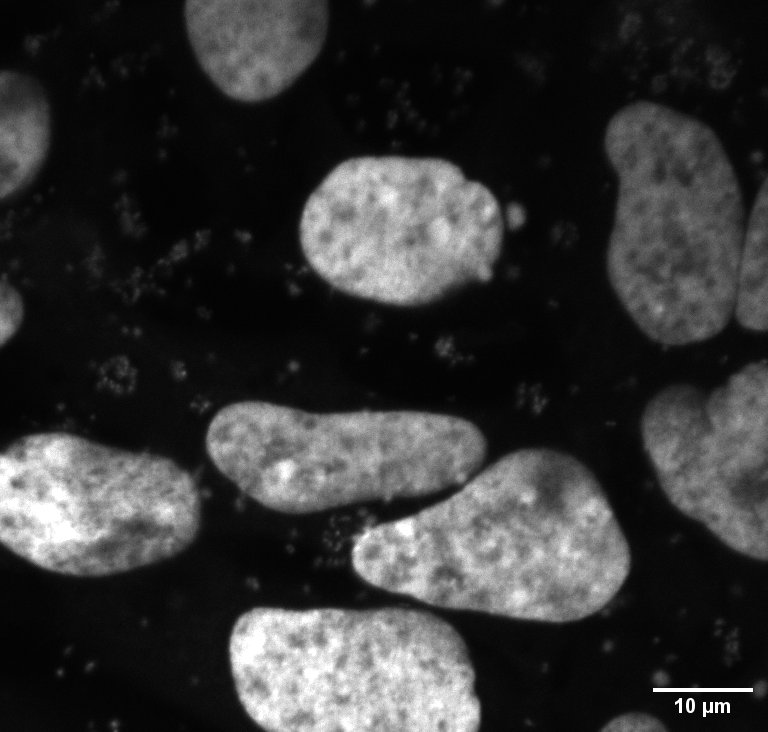

Supplement: Supplementary file 18 — Figure EV4 Source Data [file 44319_2025_674_MOESM18_ESM.zip › Figure EV4/E/B1 (SHP2-TurboID, unstimulated)/B1_F010_Z5_DAPI.jpg]

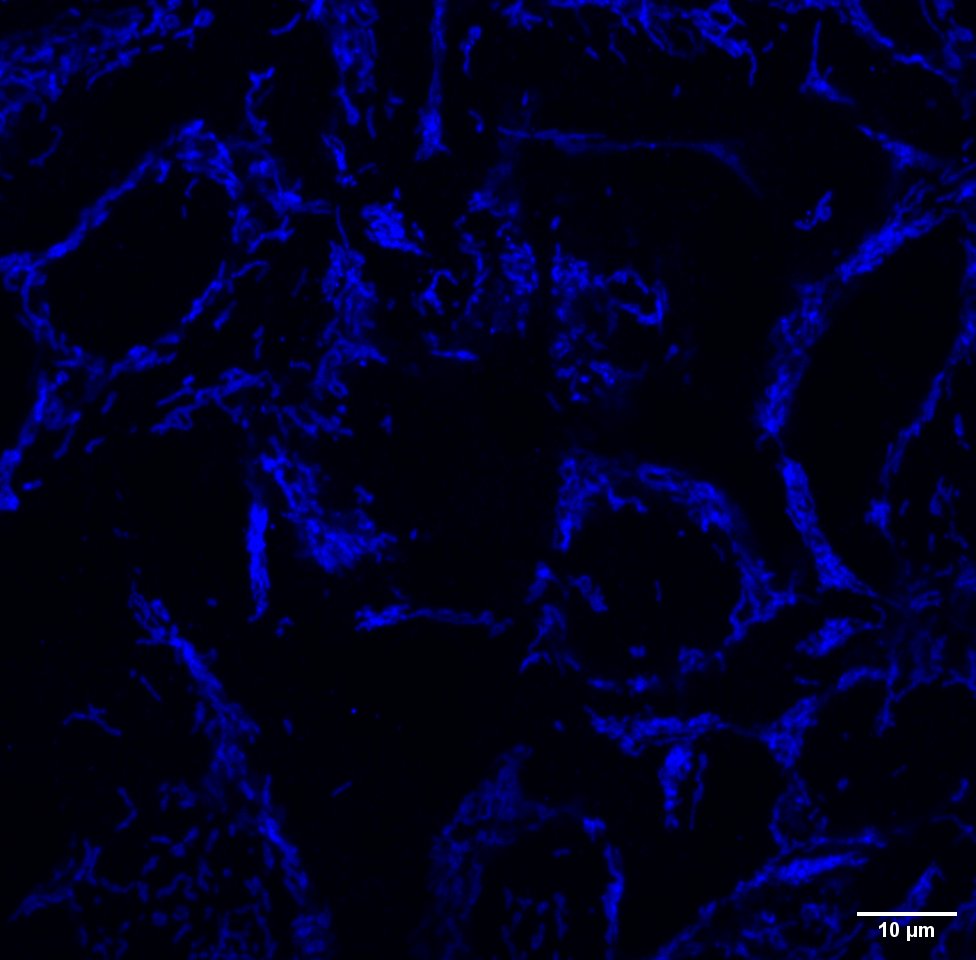

Supplement: Supplementary file 18 — Figure EV4 Source Data [file 44319_2025_674_MOESM18_ESM.zip › Figure EV4/E/A3 (SHP2-WT, unstimulated)/A3_F005_Z6_Mito.jpg]

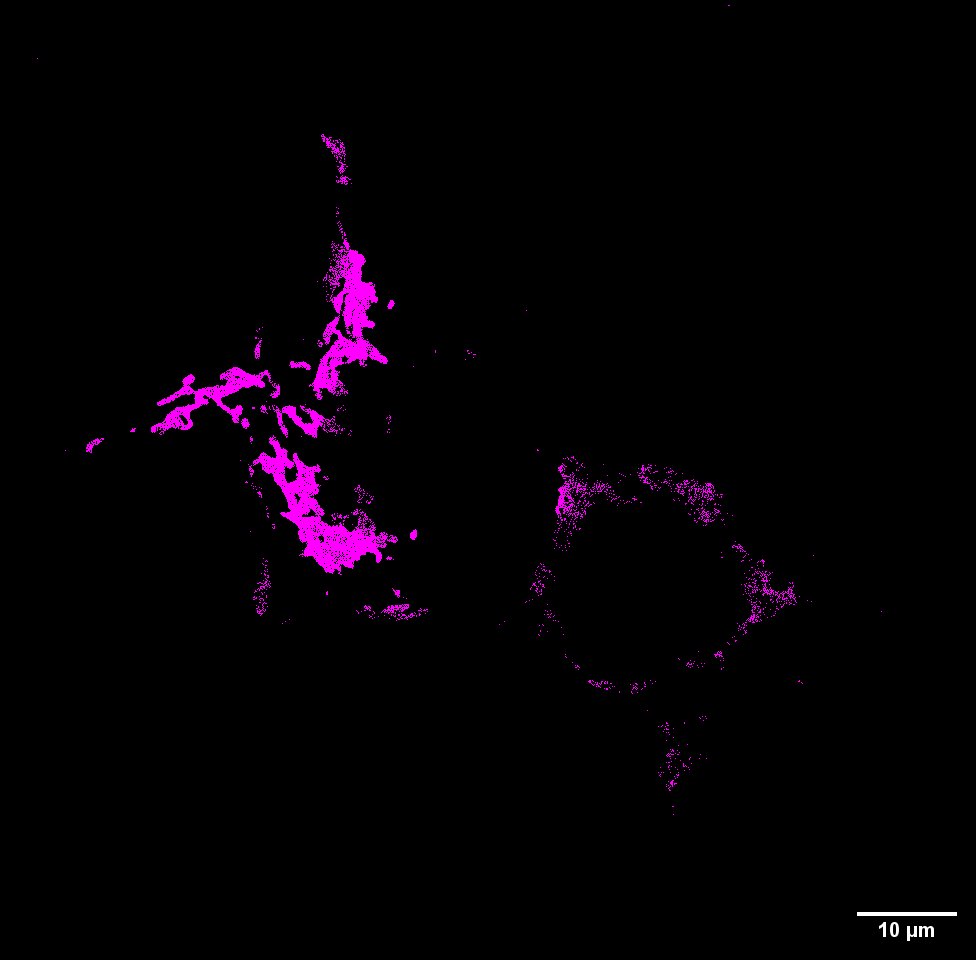

Supplement: Supplementary file 18 — Figure EV4 Source Data [file 44319_2025_674_MOESM18_ESM.zip › Figure EV4/E/A3 (SHP2-WT, unstimulated)/A3_F005_Z6_Overlap_SHP2_Mito.jpg]

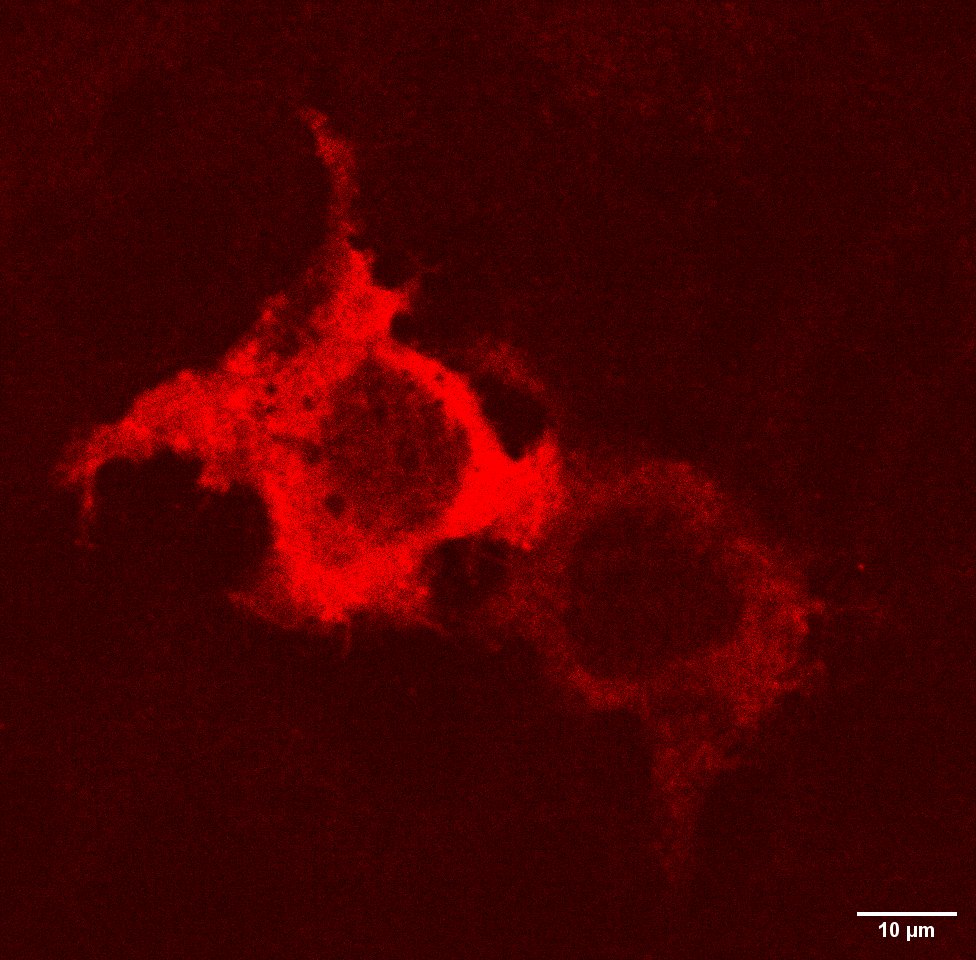

Supplement: Supplementary file 18 — Figure EV4 Source Data [file 44319_2025_674_MOESM18_ESM.zip › Figure EV4/E/A3 (SHP2-WT, unstimulated)/A3_F005_Z6_SHP2.jpg]

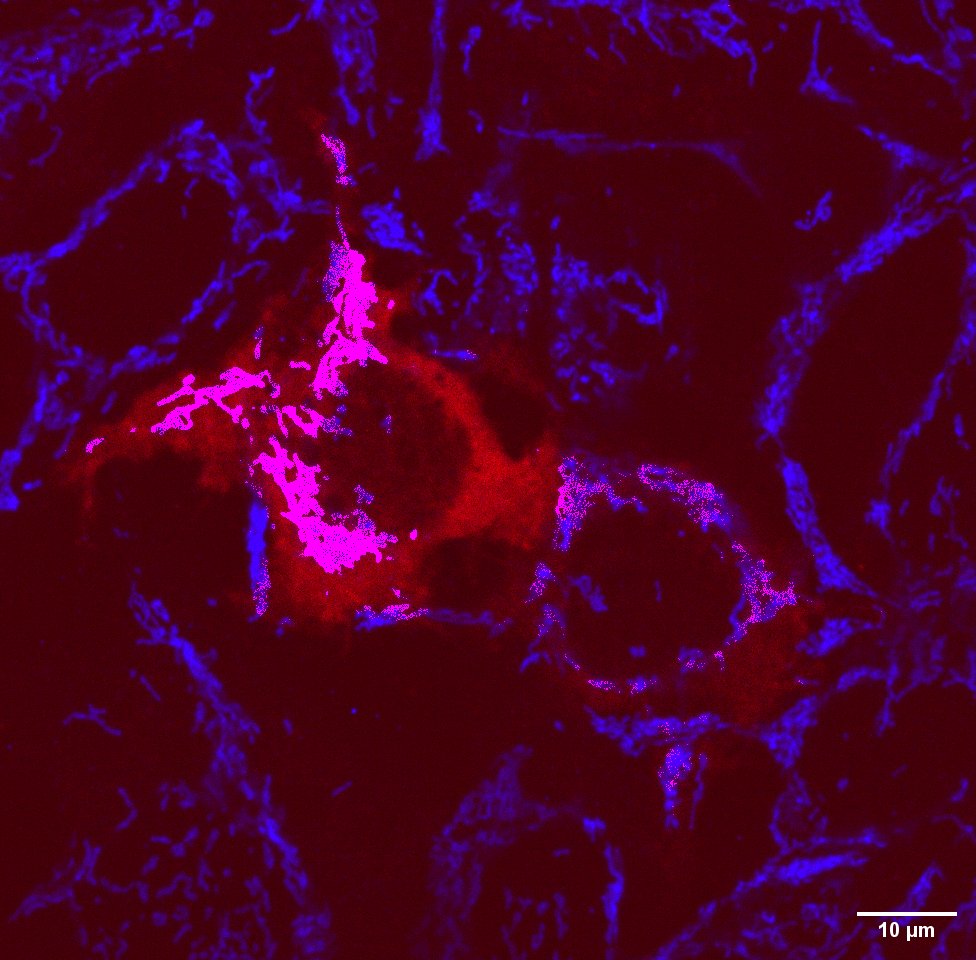

Supplement: Supplementary file 18 — Figure EV4 Source Data [file 44319_2025_674_MOESM18_ESM.zip › Figure EV4/E/A3 (SHP2-WT, unstimulated)/A3_F005_Z6_Overlay_SHP2_Mito.jpg]

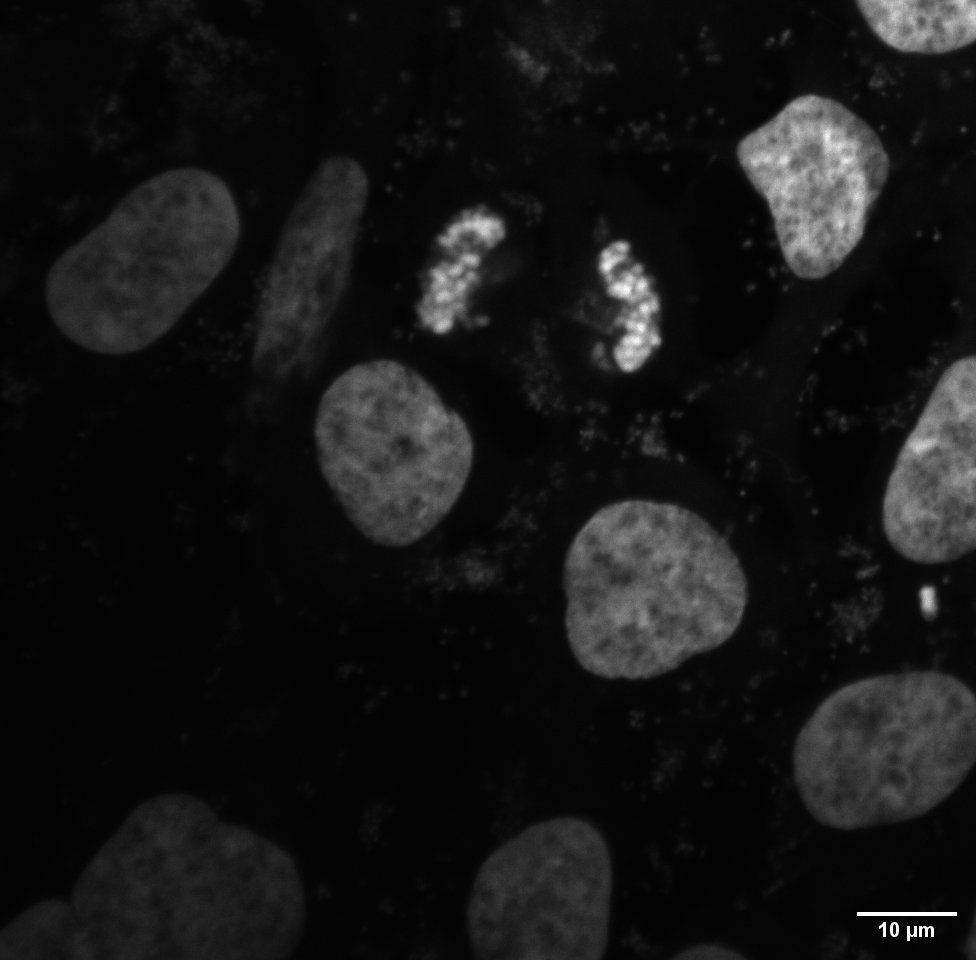

Supplement: Supplementary file 18 — Figure EV4 Source Data [file 44319_2025_674_MOESM18_ESM.zip › Figure EV4/E/A3 (SHP2-WT, unstimulated)/A3_F005_Z6_DAPI.jpg]

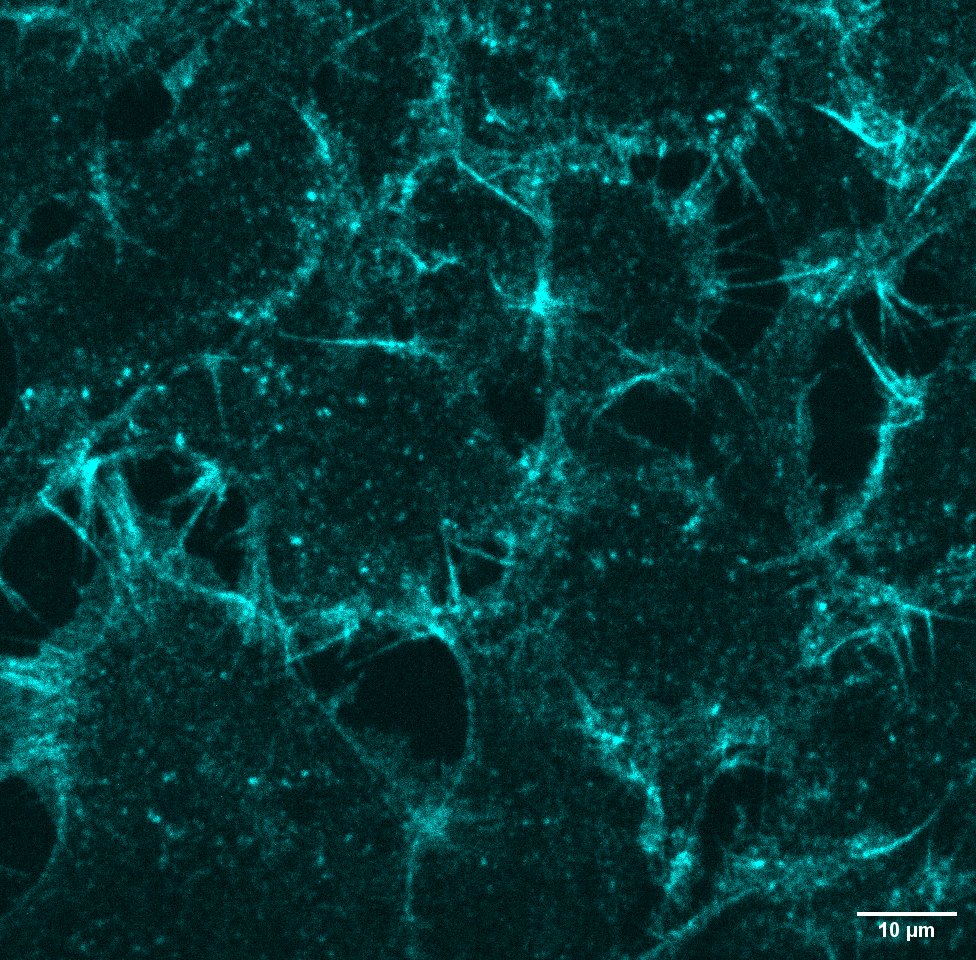

Supplement: Supplementary file 18 — Figure EV4 Source Data [file 44319_2025_674_MOESM18_ESM.zip › Figure EV4/E/A3 (SHP2-WT, unstimulated)/A3_F005_Z6_Phalloidin.jpg]

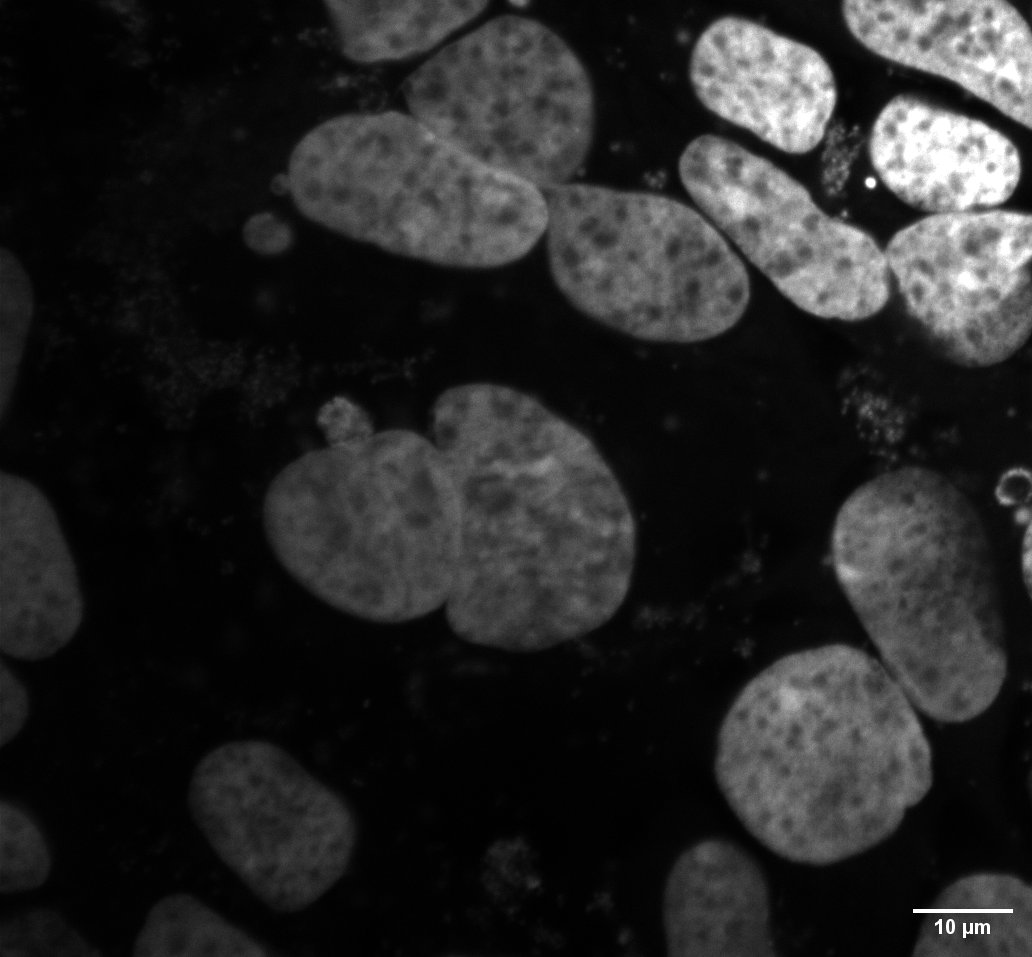

Supplement: Supplementary file 18 — Figure EV4 Source Data [file 44319_2025_674_MOESM18_ESM.zip › Figure EV4/E/A4 (SHP2-TurboID, stimulated)/A4_F005_DAPI.jpg]

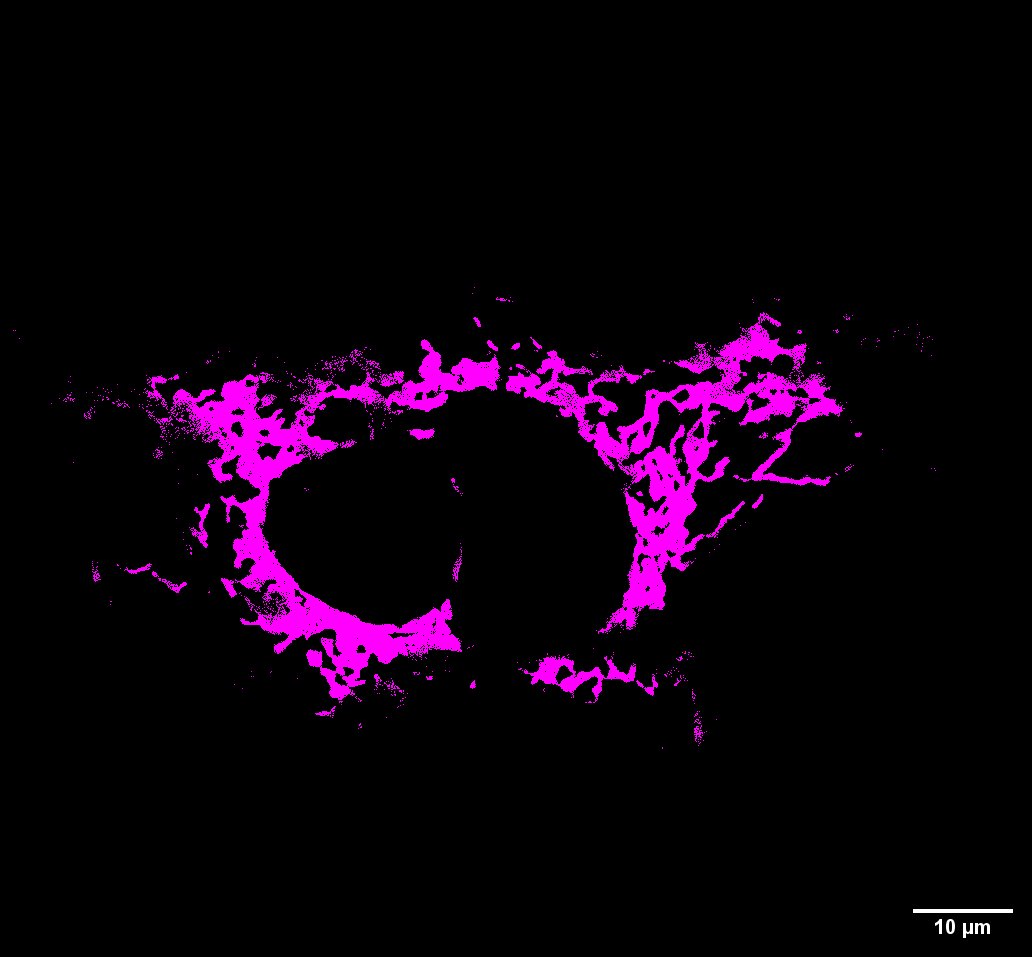

Supplement: Supplementary file 18 — Figure EV4 Source Data [file 44319_2025_674_MOESM18_ESM.zip › Figure EV4/E/A4 (SHP2-TurboID, stimulated)/A4_F005_Z7_Overlap_SHP2_Mito.jpg]

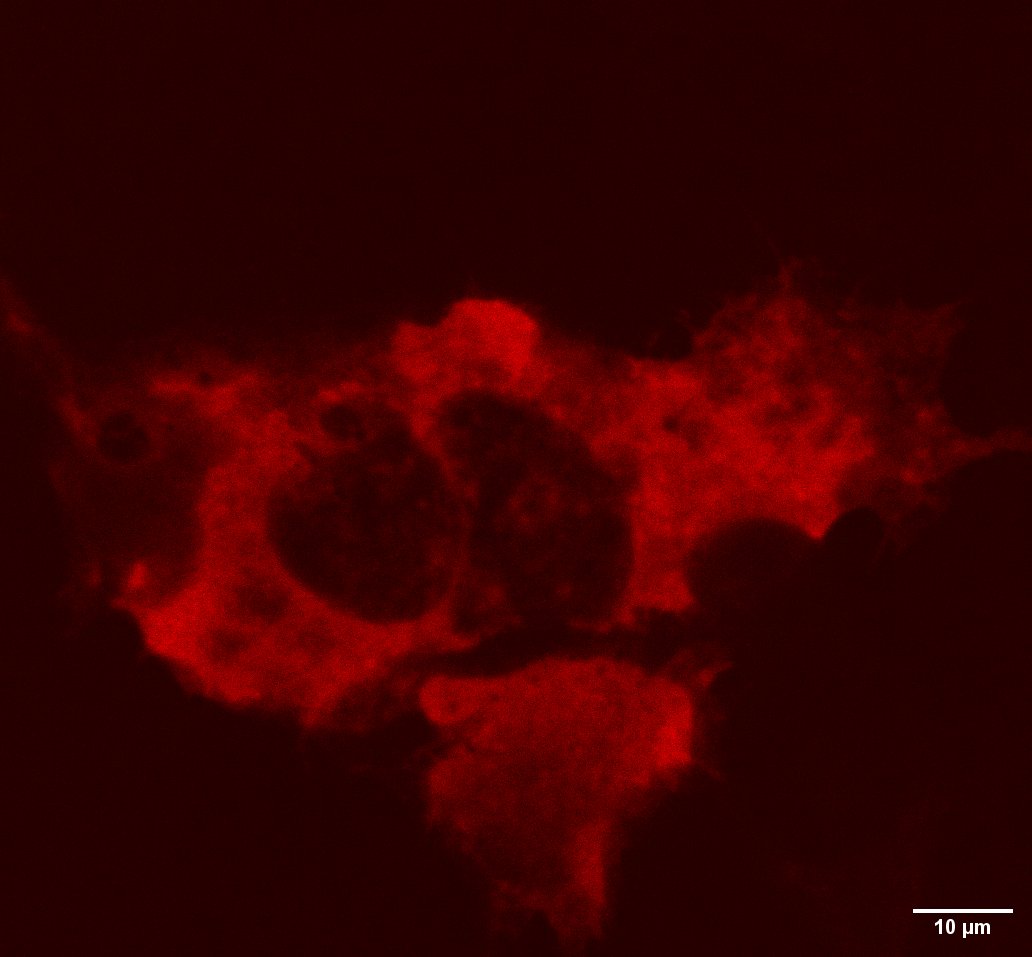

Supplement: Supplementary file 18 — Figure EV4 Source Data [file 44319_2025_674_MOESM18_ESM.zip › Figure EV4/E/A4 (SHP2-TurboID, stimulated)/A4_F005_Z7_SHP2.jpg]

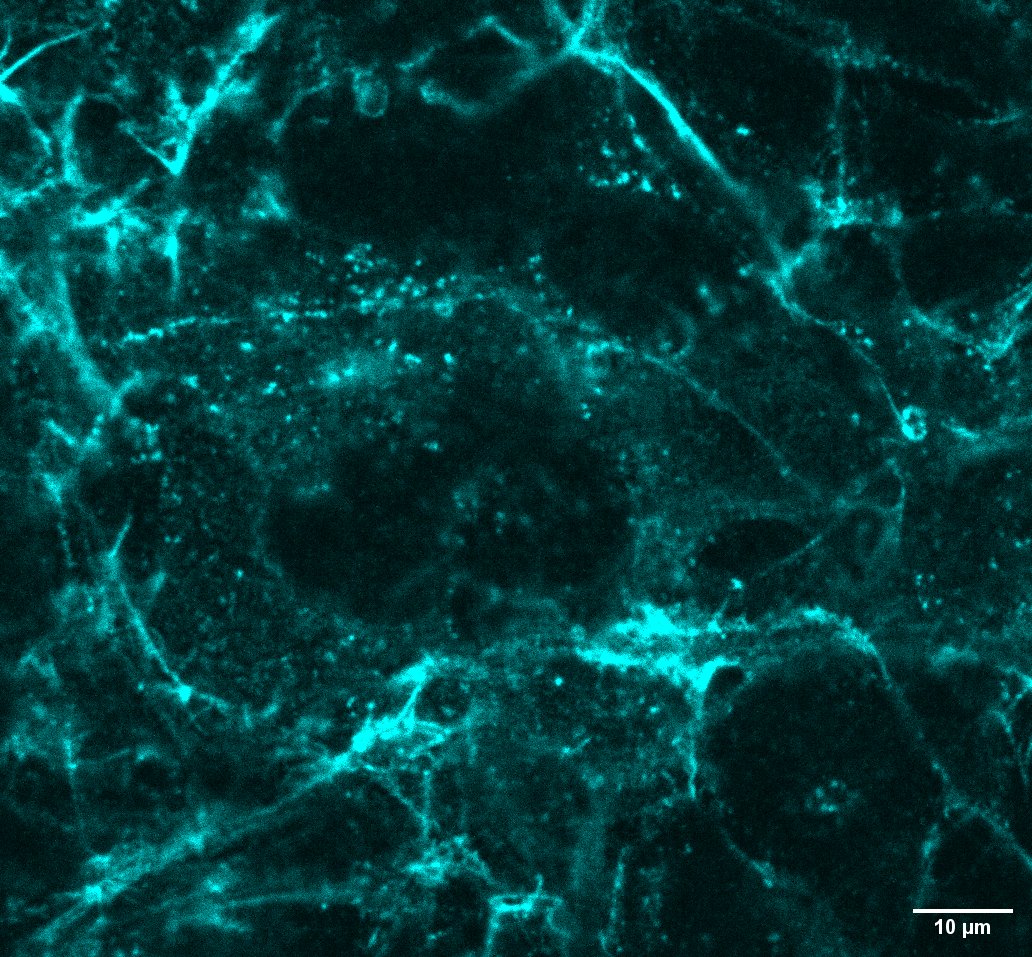

Supplement: Supplementary file 18 — Figure EV4 Source Data [file 44319_2025_674_MOESM18_ESM.zip › Figure EV4/E/A4 (SHP2-TurboID, stimulated)/A4_F005_Z7_Phalloidin.jpg]

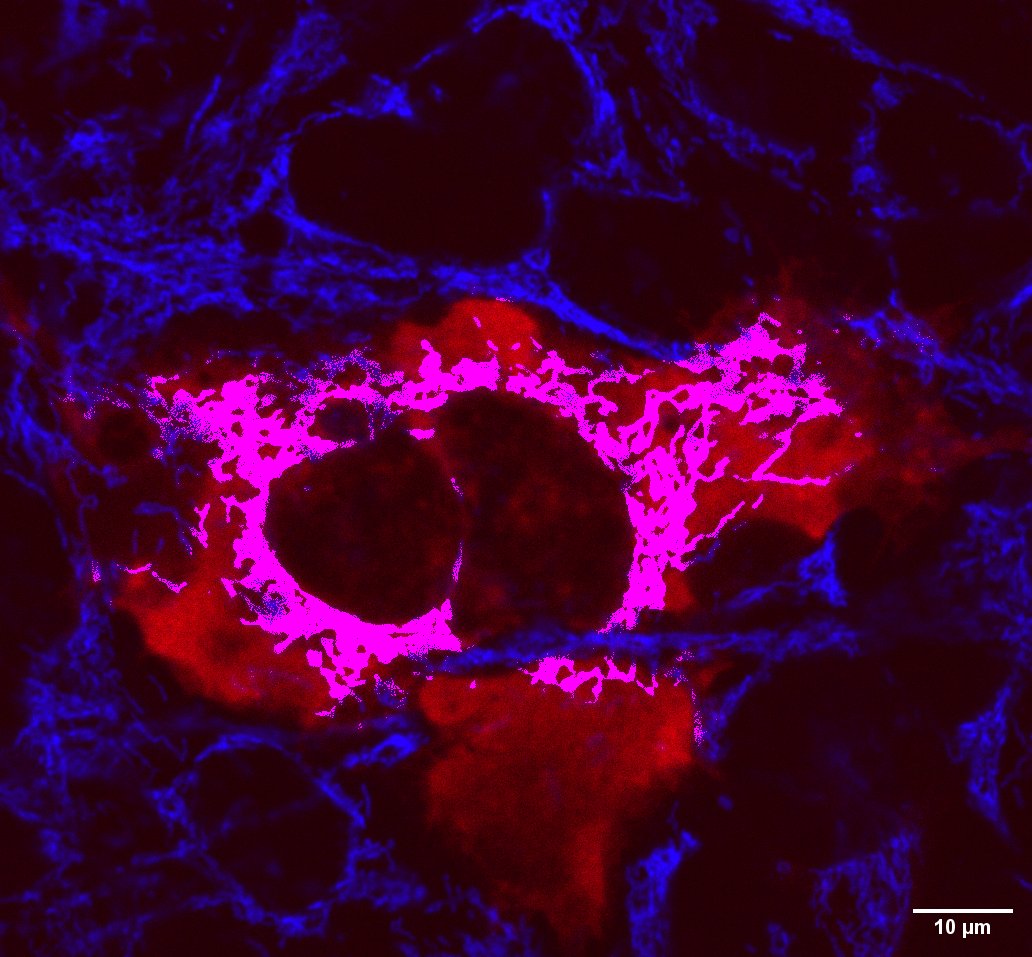

Supplement: Supplementary file 18 — Figure EV4 Source Data [file 44319_2025_674_MOESM18_ESM.zip › Figure EV4/E/A4 (SHP2-TurboID, stimulated)/A4_F005_Z7_Overlay_SHP2_Mito.jpg]

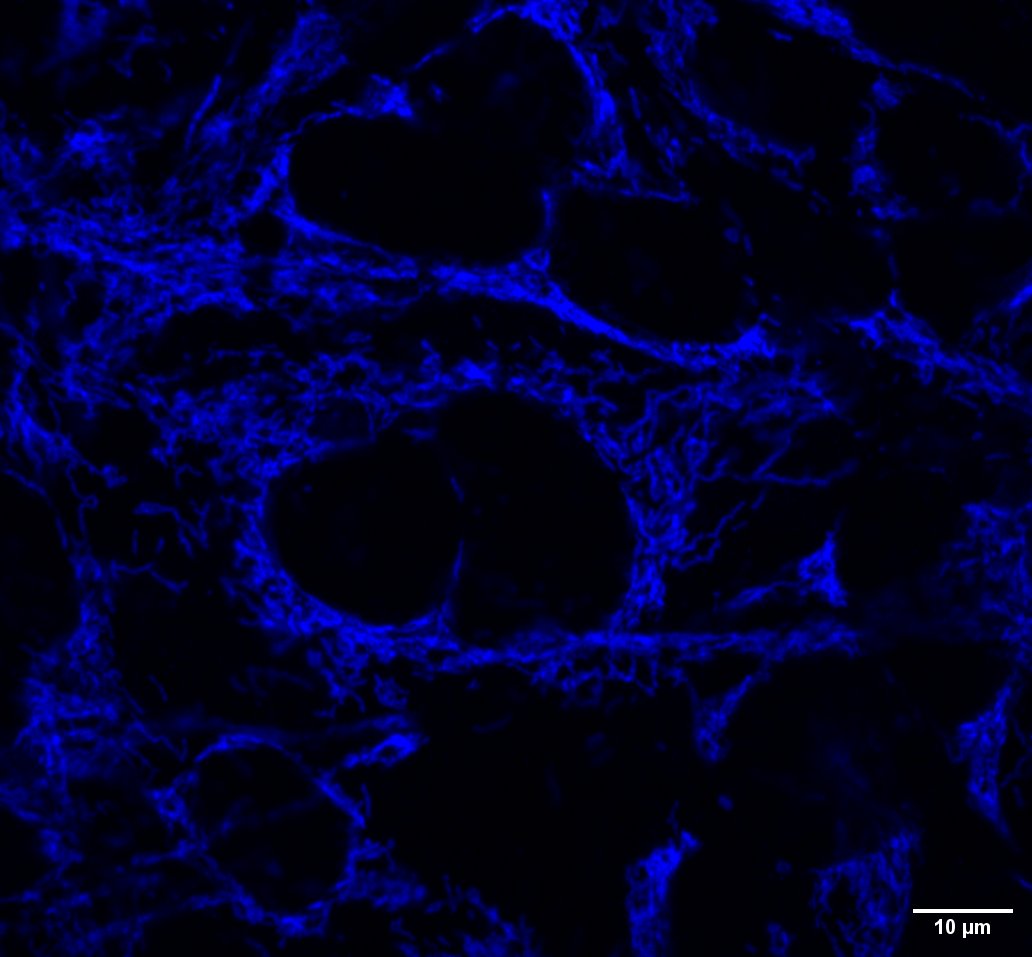

Supplement: Supplementary file 18 — Figure EV4 Source Data [file 44319_2025_674_MOESM18_ESM.zip › Figure EV4/E/A4 (SHP2-TurboID, stimulated)/A4_F005_Z7_Mito.jpg]

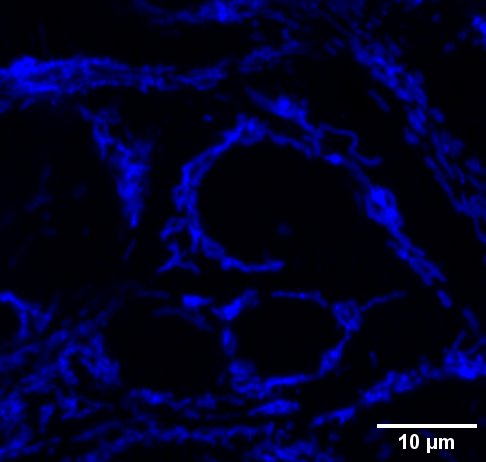

Supplement: Supplementary file 18 — Figure EV4 Source Data [file 44319_2025_674_MOESM18_ESM.zip › Figure EV4/E/A2 (SHP2-WT, stimulated)/A2_F040_Z5_mito.jpg]

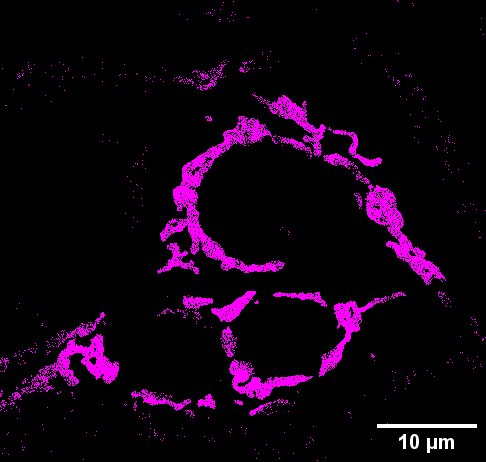

Supplement: Supplementary file 18 — Figure EV4 Source Data [file 44319_2025_674_MOESM18_ESM.zip › Figure EV4/E/A2 (SHP2-WT, stimulated)/A2_F040_Z5_Overlap_SHP2_mito.jpg]

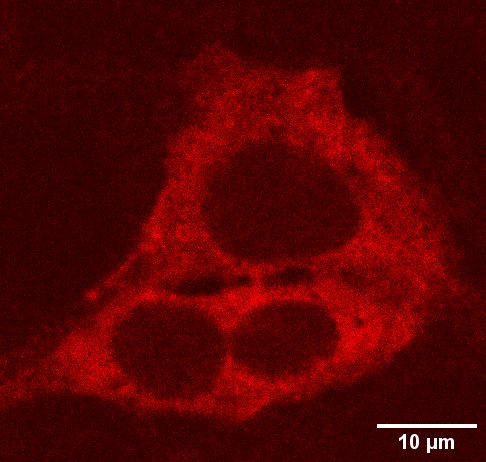

Supplement: Supplementary file 18 — Figure EV4 Source Data [file 44319_2025_674_MOESM18_ESM.zip › Figure EV4/E/A2 (SHP2-WT, stimulated)/A2_F040_Z5_SHP2.jpg]

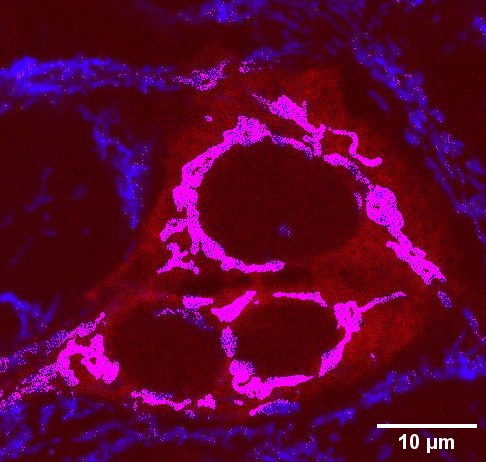

Supplement: Supplementary file 18 — Figure EV4 Source Data [file 44319_2025_674_MOESM18_ESM.zip › Figure EV4/E/A2 (SHP2-WT, stimulated)/A2_F040_Z5_Overlay_SHP2_mito.jpg]

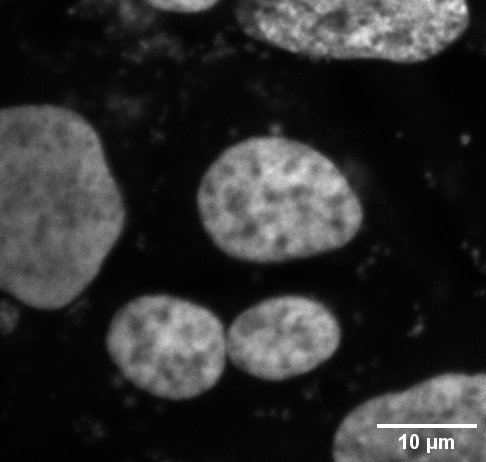

Supplement: Supplementary file 18 — Figure EV4 Source Data [file 44319_2025_674_MOESM18_ESM.zip › Figure EV4/E/A2 (SHP2-WT, stimulated)/A2_F040_Z5_DAPI.jpg]

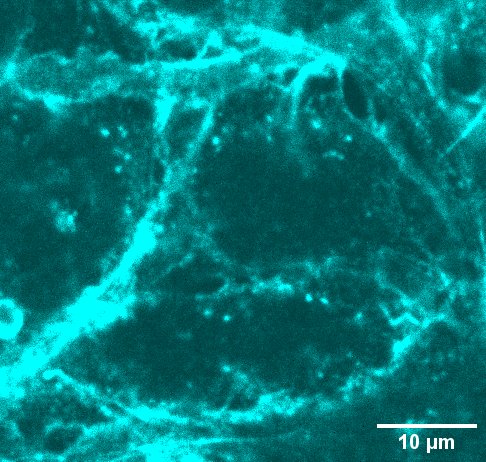

Supplement: Supplementary file 18 — Figure EV4 Source Data [file 44319_2025_674_MOESM18_ESM.zip › Figure EV4/E/A2 (SHP2-WT, stimulated)/A2_F040_Z5_Phallodin.jpg]
