# Supplementary material for: Proximity-labeling proteomics reveals remodeled interactomes and altered localization of pathogenic SHP2 variants
Source: EMBO Rep. 2025 Dec 22;27(3):793–826. doi: 10.1038/s44319-025-00674-4 (PMC12894930; doi:10.1038/s44319-025-00674-4)
Supplement: Supplementary file 25 — Expanded View Figures [file 44319_2025_674_MOESM25_ESM.pdf]

Expanded View Figures

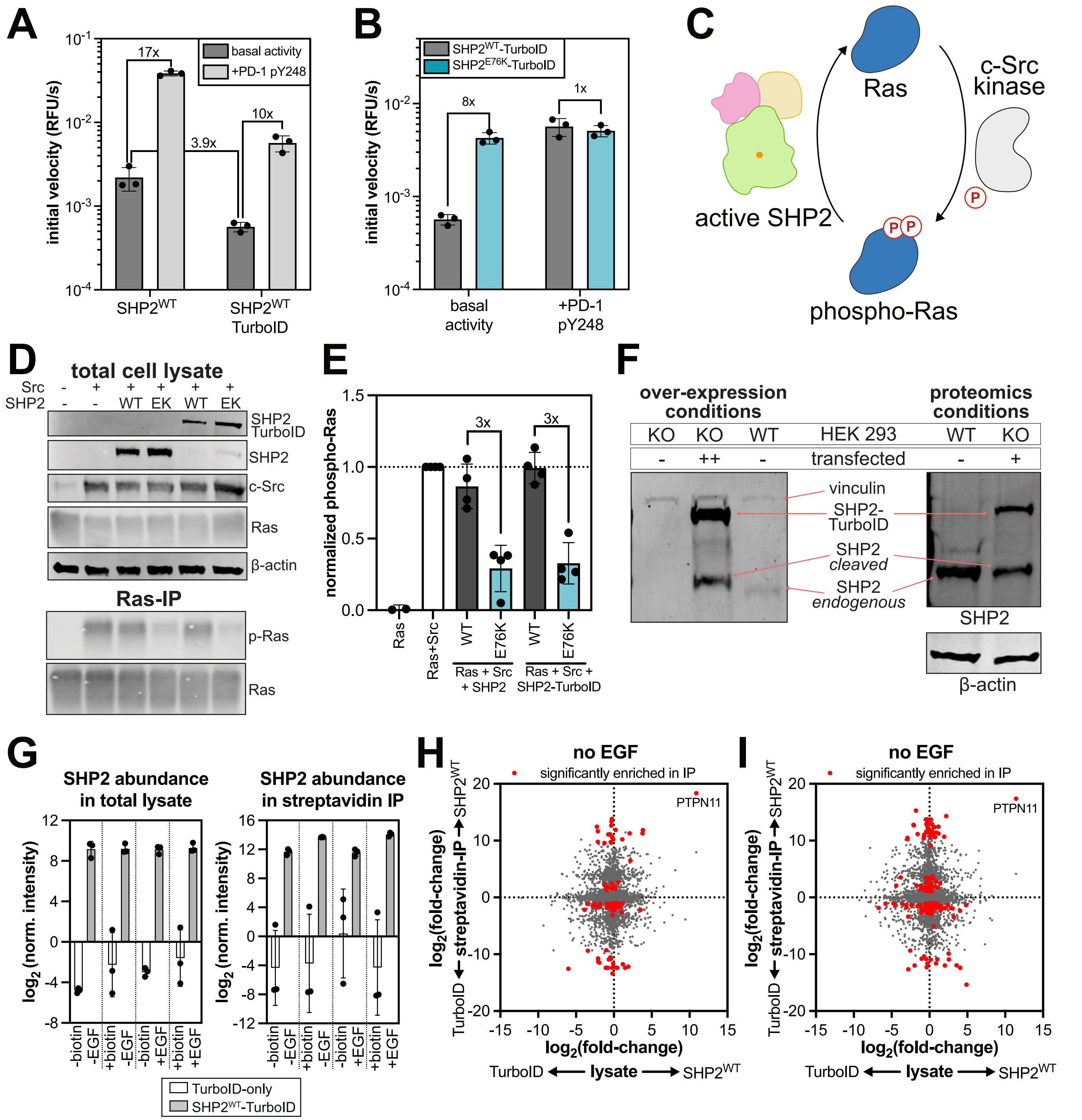

 © The Author(s)

Figure EV1. Quality control and validation of the SHP2-TurboID system.

(**A**) In vitro activity measurements showing that SHP2-TurboID can be activated by a phosphopeptide (PD-1 pY248) ($n = 3$). Bar heights indicate the mean, and error bars indicate standard deviation. (**B**) In vitro activity measurements showing that E76K hyperactivation is preserved in a TurboID-fusion context, and only SHP2^{WT}-TurboID, but not SHP2^{E76K}-TurboID can be further activated by PD-1 pY248 ($n = 3$). Bar heights indicate the mean, and error bars indicate standard deviation. (**C**) Schematic of the Ras dephosphorylation assay in HEK 293 cells. (**D**) Representative western blots showing dephosphorylation of N-Ras by SHP2-TurboID proteins. WT = SHP2^{WT} EK = SHP2^{E76K}. (**E**) Quantification of Ras dephosphorylation assays ($n = 4$). Bar heights indicate the mean, and error bars indicate standard deviation. (**F**) The left blot compares SHP2 levels in WT and SHP2^{KO} HEK 293 cells, and shows over-expression of SHP2^{WT}-TurboID in SHP2^{KO} cells, highlighting partial cleavage of SHP2-TurboID. The right blot compares endogenous SHP2 levels in WT HEK 293 cells with SHP2^{WT}-TurboID levels in SHP2^{KO} HEK 293 cells, transfected under the same conditions used for proteomics experiments. (**G**) Further confirmation of SHP2^{KO} cells used for proteomics experiments, comparing SHP2 levels detected by mass spectrometry in negative control TurboID-only samples and SHP2^{WT}-TurboID samples. Bar heights indicate the mean, and error bars indicate standard deviation. (**H**) Fold-change between SHP2^{WT}-TurboID and TurboID-only for total lysates, measuring protein abundance (x-axis), and streptavidin-IP, measuring proximity labeling (y-axis), in unstimulated cells. (**I**) Same as (**H**), but for cells stimulated with 100 ng/mL EGF ($n = 3$ for total lysate and TurboID datasets). Source data are available online for this figure.

© The Author(s)

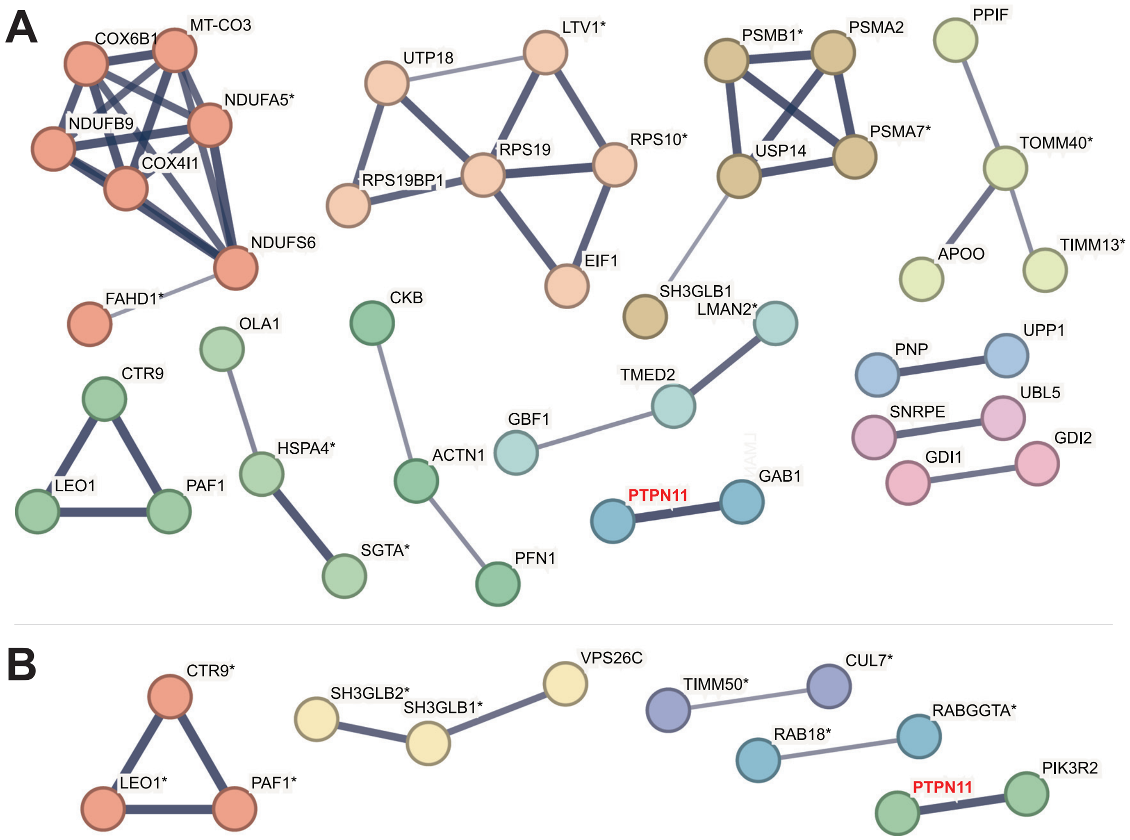

Figure EV2. STRING interaction networks for SHP2^{WT}-TurboID hits.

(**A**) STRING interaction network for proteins enriched by SHP2^{WT}-TurboID over the TurboID control by at least twofold with a *p* value <0.1 (heteroscedastic, unpaired *t*-test), in the absence of EGF stimulation. (**B**) Same as in (**A**) but with EGF stimulation. Proteins with a *p* value between 0.05 and 0.1 are marked with an asterisk. All other proteins have a *p* value <0.05. For both panels, solid lines between proteins indicate a known physical interaction, as documented in the STRING database. Only proteins that have a physical interaction with at least one other protein in our interactomes and have an edge confidence score of at least 0.4 are shown. Edge thickness represents edge confidence: thin = 0.4, medium = 0.7, thick = 0.9. Clusters were identified by Markov Clustering (MCL). Source data are available online for this figure.

 © The Author(s)

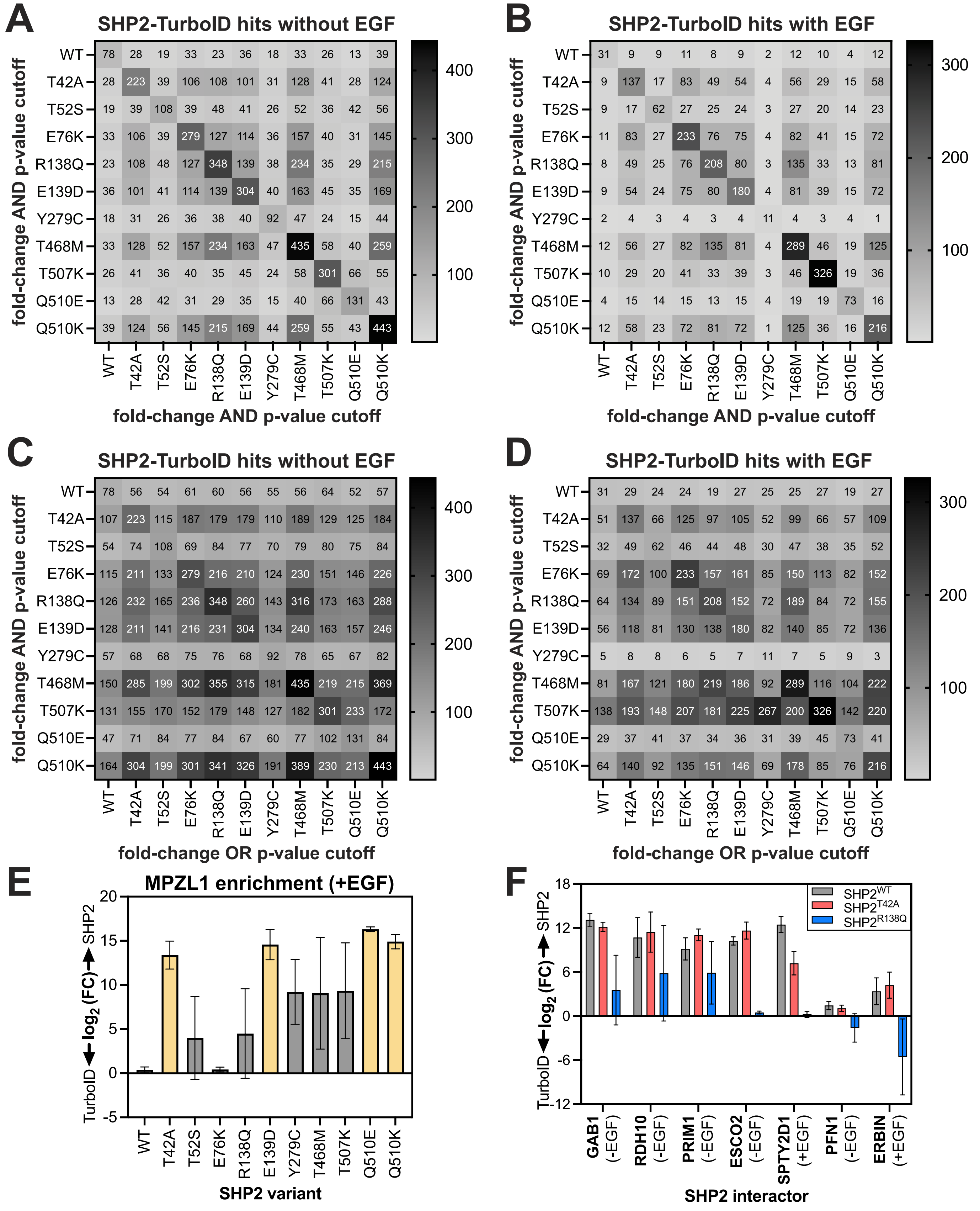
A
SHP2-TurboID hits without EGF
fold-change AND p-value cutoff
B
SHP2-TurboID hits with EGF
fold-change AND p-value cutoff
C
SHP2-TurboID hits without EGF
fold-change AND p-value cutoff
fold-change OR p-value cutoff
D
SHP2-TurboID hits with EGF
fold-change AND p-value cutoff
fold-change OR p-value cutoff
WT T42A T52S E76K R138Q E139D Y279C T468M T507K Q510E Q510K
E
MPZL1 enrichment (+EGF)
TurboID ← log2(FC) → SHP2
SHP2 variant
F
SHP2WT
SHP2T42A
SHP2R138Q
GAB1 (-EGF)
RDH10 (-EGF)
PRIM1 (-EGF)
ESCO2 (-EGF)
SPTY2D1 (+EGF)
PFN1 (-EGF)
ERBIN (+EGF)
SHP2 interactor

Figure EV3. Mutation- and EGF-dependent changes in SHP2-TurboID proximity labeling.

(**A**) Stringent pairwise overlap in hits for all SHP2 variants relative to the TurboID control in unstimulated samples. Hits are defined as proteins with >2-fold enrichment and a *p* value <0.05 (heteroscedastic, unpaired *t*-test), and overlap indicates the number of proteins that meet these criteria for both SHP2 variants in the comparison. (**B**) Same as in (**A**) but with EGF stimulation. (**C**) Relaxed pairwise overlap in hits for all SHP2 variants relative to TurboID control in unstimulated samples. For SHP2 variants on the y-axis, a hit was defined as proteins with both >2-fold enrichment and a *p* value <0.05. For SHP2 variants on the x-axis, a hit was defined as proteins with either >2-fold enrichment or a *p* value <0.05. (**D**) Same as (**C**), but with EGF stimulation. Note that in panels (**A–D**), the smaller numbers for $SHP2^{Y279C}$ reflect the use of only two replicates for this mutant, due to an instrument error and sample loss. (**E**) Enrichment of MPZL1 in our dataset for all SHP2 variants relative to the TurboID-only control (+EGF), highlighting significant proximity labeling with some mutants ($n = 3$ biological replicates for all samples except $SHP2^{Y279C}$, which had two biological replicates). Yellow indicates a significant difference from the TurboID-only control (fold-change >2, *p* value <0.05 from a heteroscedastic, unpaired *t*-test): $p = 0.005656$ (T42A), $p = 0.00580$ (E139D), $p = 0.00003$ (Q510E), and $p = 0.00049$ (Q510K). (**F**) Enrichment of proteins with predicted C-SH2 binding sites (top ~30th percentile) by $SHP2^{WT}$, $SHP2^{T42A}$, and $SHP2^{R138Q}$, showing loss of signal for C-SH2-dead $SHP2^{R138Q}$ ($n = 3$ biological replicates). In panels (**E**, **F**), bar heights indicate the mean, and error bars indicate standard deviation. Source data are available online for this figure.

 © The Author(s)

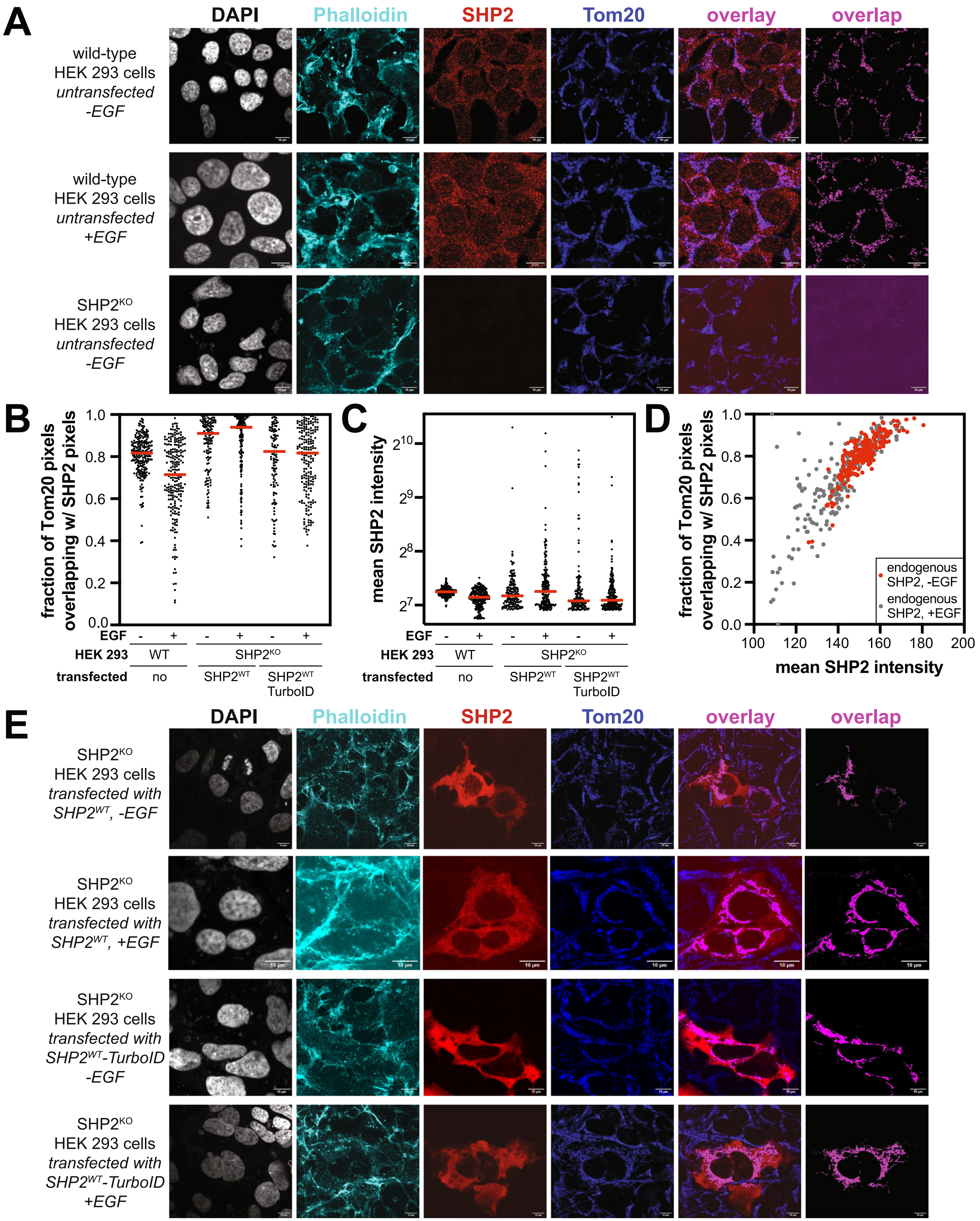
A
DAPI
Phalloidin
SHP2
Tom20
overlay
overlap
wild-type HEK 293 cells untransfected -EGF
wild-type HEK 293 cells untransfected +EGF
SHP2KO HEK 293 cells untransfected -EGF
B
fraction of Tom20 pixels overlapping w/ SHP2 pixels
1.0
0.8
0.6
0.4
0.2
0.0
EGF - + - + - +
HEK 293 WT SHP2KO
transfected no SHP2WT SHP2WT TurboID
C
mean SHP2 intensity
2^10
2^9
2^8
2^7
EGF - + - + - +
HEK 293 WT SHP2KO
transfected no SHP2WT SHP2WT TurboID
D
fraction of Tom20 pixels overlapping w/ SHP2 pixels
endogenous SHP2, -EGF
endogenous SHP2, +EGF
100 120 140 160 180 200
mean SHP2 intensity
E
DAPI
Phalloidin
SHP2
Tom20
overlay
overlap
SHP2KO HEK 293 cells transfected with SHP2WT, -EGF
SHP2KO HEK 293 cells transfected with SHP2WT, +EGF
SHP2KO HEK 293 cells transfected with SHP2WT-TurboID -EGF
SHP2KO HEK 293 cells transfected with SHP2WT-TurboID +EGF

© The Author(s)

Figure EV4. Analysis of SHP2 localization by fluorescence microscopy.

(**A**) Representative images of endogenous SHP2 localization by confocal fluorescence microscopy showing co-localization of SHP2 with mitochondria in wild-type HEK 293 cells, and absence of SHP2 in $SHP2^{KO}$ HEK 293 cells. (**B**) Fraction of Tom20-positive pixels that overlap with SHP2-positive pixels. (**C**) Mean fluorescence intensity of all SHP2-positive pixels in cells used to assess the mitochondrial overlap in panel (**B**). In panels (**B**, **C**), the red lines are median values, $N = 129$–234 cells, and the $SHP2^{KO}$ cells $-/+$ EGF have a statistically significant difference in both co-localization and mean SHP2 intensity ($p < 0.0001$, Welch's unpaired *t*-test). (**D**) Correlation between mean SHP2 intensity in cells and SHP2 pixel overlap with Tom20. (**E**) Representative images of SHP2 localization in $SHP2^{KO}$ HEK 293 cells transfected with different SHP2 constructs, unstimulated or stimulated with EGF. Source data are available online for this figure.

 © The Author(s)

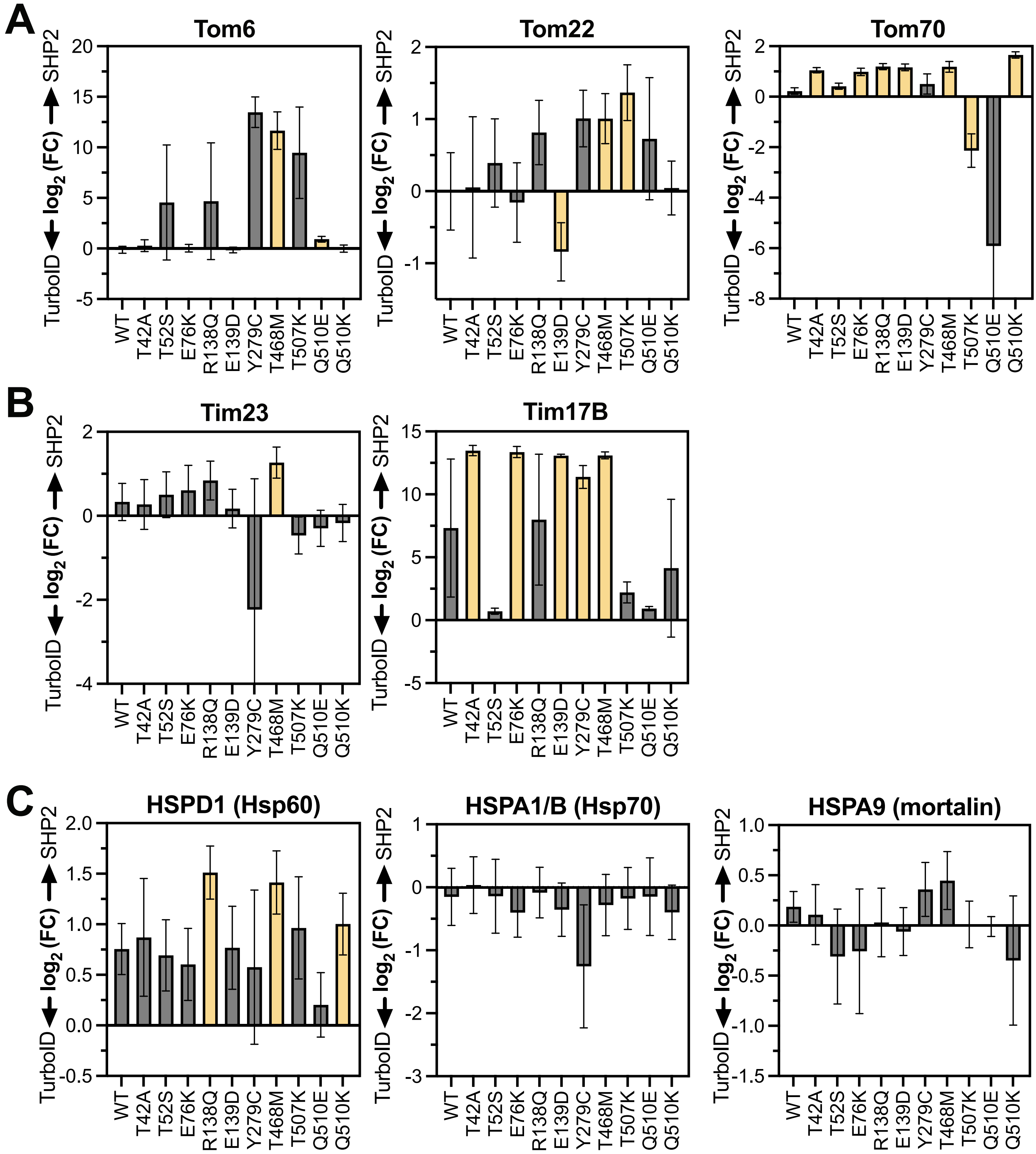
A
Tom6
Tom22
Tom70
B
Tim23
Tim17B
C
HSPD1 (Hsp60)
HSPA1/B (Hsp70)
HSPA9 (mortalin)
TurboID ← log2 (FC) → SHP2
WT
T42A
T52S
E76K
R138Q
E139D
Y279C
T468M
T507K
Q510E
Q510K

© The Author(s)

Figure EV5. Mutant-specific labeling of mitochondrial import proteins and chaperones.

In all panels, yellow bars indicate a significant difference from the TurboID-only control (fold-change >2, p value <0.05 from a heteroscedastic, unpaired t-test). Bar heights indicate the mean, and error bars indicate standard deviation. (**A**) Enrichment or depletion of select Tom proteins in our dataset for all SHP2 variants relative to the TurboID-only control. For Tom6, $p = 0.01094$ (T468M) and $p = 0.02227$ (Q510E). For Tom22, $p = 0.04200$ (E139D), $p = 0.01760$ (T468M), and $p = 0.00753$ (T507K). For Tom70, $p = 0.00051$ (T42A), $p = 0.00860$ (T52S), $p = 0.00092$ (E76K), $p = 0.00017$ (R138Q), $p = 0.00025$ (E139D), $p = 0.00537$ (T468M), $p = 0.04238$ (T507K), and $p = 5.06401 \times 10^{-5}$ (Q510K). (**B**) Same as (**A**), but for two components of the Tim23 complex in our dataset. For Tim23, $p = 0.02098$ (T468M). For Tim17B, $p = 0.00019$ (T42A), $p = 0.00026$ (E76K), $p = 1.28078 \times 10^{-5}$ (E139D), $p = 0.04912$ (Y279C), $p = 1.9238 \times 10^{-5}$ (T468M). (**C**) Same as (**A**) but for the mitochondrial chaperonin HSPD1/Hsp60, HSPA1A/HSPA1B (cytosolic Hsp70), and HSPA9/mortalin (mitochondrial Hsp70). For HSPD1/Hsp60, $p = 0.00509$ (R138Q), $p = 0.00339$ (T468M), and $p = 0.01100$ (Q510K). Source data are available online for this figure.

© The Author(s)
